# A physical map of the bovine genome

**DOI:** 10.1186/gb-2007-8-8-r165

**Published:** 2007-08-14

**Authors:** Warren M Snelling, Readman Chiu, Jacqueline E Schein, Matthew Hobbs, Colette A Abbey, David L Adelson, Jan Aerts, Gary L Bennett, Ian E Bosdet, Mekki Boussaha, Rudiger Brauning, Alexandre R Caetano, Marcos M Costa, Allan M Crawford, Brian P Dalrymple, André Eggen, Annelie Everts-van der Wind, Sandrine Floriot, Mathieu Gautier, Clare A Gill, Ronnie D Green, Robert Holt, Oliver Jann, Steven JM Jones, Steven M Kappes, John W Keele, Pieter J de Jong, Denis M Larkin, Harris A Lewin, John C McEwan, Stephanie McKay, Marco A Marra, Carrie A Mathewson, Lakshmi K Matukumalli, Stephen S Moore, Brenda Murdoch, Frank W Nicholas, Kazutoyo Osoegawa, Alice Roy, Hanni Salih, Laurent Schibler, Robert D Schnabel, Licia Silveri, Loren C Skow, Timothy PL Smith, Tad S Sonstegard, Jeremy F Taylor, Ross Tellam, Curtis P Van Tassell, John L Williams, James E Womack, Natasja H Wye, George Yang, Shaying Zhao, the International Bovine BAC Mapping Consortium

**Affiliations:** 1 USDA, ARS, US Meat Animal Research Center, Clay Center, NE 68933, USA; 2 Genome Sciences Centre, British Columbia Cancer Agency, Vancouver, BC, Canada; 3 Cooperative Research Centre for Innovative Dairy Products, Reprogen, Faculty of Veterinary Science, University of Sydney, NSW 2006, Australia; 4 Texas A&M University, College Station, TX 77843, USA; 5 Roslin Institute, Roslin, Midlothian EH25 9PS, UK; 6 INRA, UR339 Laboratoire de Génétique Biochimique et de Cytogénétique, 78350 Jouy-en-Josas, France; 7 AgResearch, Invermay, Mosgiel, New Zealand; 8 Embrapa Recursos Geneticos e Biotecnologia, Parque Estacao Biologica, Final Av. W/5 Norte, Brasilia-DF, CP 02372 70770-900, Brasil; 9 CSIRO Livestock Industries, Carmody Road, St Lucia, Queensland 4067, Australia; 10 Department of Animal Sciences, University of Illinois at Urbana-Champaign, Urbana, IL 61801, USA; 11 USDA-ARS - National Program Staff, Beltsville, MD 20705-5134, USA; 12 Children's Hospital Oakland Research Institute, Oakland, California 94609, USA; 13 Institute for Genomic Biology, University of Illinois at Urbana-Champaign, Urbana, IL 61801, USA; 14 Department of Agricultural, Food and Nutritional Science, University of Alberta, Edmonton, Alberta T6G 2P5, Canada; 15 USDA, ARS, BARC Bovine Functional Genomics Laboratory, Maryland, USA; 16 Genoscope, rue Gaston Cremieux, 91057 Evry, France; 17 Animal Science Research Center, Division of Animal Sciences, University of Missouri, Columbia, MO 65211, USA; 18 Istituto di Zootecnica Università Cattolica del S Cuore, via E Parmense, 84 29100 Piacenza, Italy; 19 Current address: Parco Tecnologico Padano, Via Einstein, Polo Universitario, Lodi 26900, Italy; 20 The Institute for Genomic Research, Rockville, Maryland 20850, USA; 21 Current address: Department of Biochemistry and Molecular Biology, University of Georgia, Green Street, Athens, GA 30602-7229, USA

## Abstract

**Background:**

Cattle are important agriculturally and relevant as a model organism. Previously described genetic and radiation hybrid (RH) maps of the bovine genome have been used to identify genomic regions and genes affecting specific traits. Application of these maps to identify influential genetic polymorphisms will be enhanced by integration with each other and with bacterial artificial chromosome (BAC) libraries. The BAC libraries and clone maps are essential for the hybrid clone-by-clone/whole-genome shotgun sequencing approach taken by the bovine genome sequencing project.

**Results:**

A bovine BAC map was constructed with *Hin*dIII restriction digest fragments of 290,797 BAC clones from animals of three different breeds. Comparative mapping of 422,522 BAC end sequences assisted with BAC map ordering and assembly. Genotypes and pedigree from two genetic maps and marker scores from three whole-genome RH panels were consolidated on a 17,254-marker composite map. Sequence similarity allowed integrating the BAC and composite maps with the bovine draft assembly (Btau3.1), establishing a comprehensive resource describing the bovine genome. Agreement between the marker and BAC maps and the draft assembly is high, although discrepancies exist. The composite and BAC maps are more similar than either is to the draft assembly.

**Conclusion:**

Further refinement of the maps and greater integration into the genome assembly process may contribute to a high quality assembly. The maps provide resources to associate phenotypic variation with underlying genomic variation, and are crucial resources for understanding the biology underpinning this important ruminant species so closely associated with humans.

## Abstract 2

A new physical map of the bovine genome has been constructed by integrating data from genetic and radiation hybrid maps, and a new bovine BAC map, with the bovine genome draft assembly.

## Background

Cattle have played a crucial role throughout recent human agrarian history, providing draft power, milk and meat for human consumption since domestication 8,000 years ago [1,2]. Cattle studies have contributed to our knowledge of endocrine function, fertilization, and growth, and enhanced our understanding of genetics, selection and evolution [3,4]. However, much remains to be determined; particularly, how cattle have adapted to intense selection pressures since domestication and how ruminants convert low quality forages into energy and protein-dense meat and milk. Worldwide, roughly 1,000 different breeds and varieties have been recognized [5]. These breeds originated in different locations, were subjected to different environments, and possess somewhat different characteristics as a consequence of ongoing natural and artificial selection. Ancestry of much of today's seedstock can be traced to breed-specific herdbooks established in the mid-1800's [2]. Formalization of the genetic selection process has culminated in extensive estimation of heritabilities, and genetic and environmental relationships between traits [6,7], coupled with objective approaches to animal evaluation [8] and selection [9]. These have been widely utilized in the development of modern beef and dairy performance recording and evaluation schemes [10-12]. The phenotype-based selection systems developed and optimized in the last century are now moving towards integration of DNA information to accelerate genetic progress.

While numerous quantitative trait loci (QTL) have been mapped [13,14], only a small number of quantitative trait nucleotides or causative mutations [15-19] have been identified for economically important cattle QTL. We expect further development of cattle genomic resources to accelerate discovery of causative mutations, and facilitate genome-wide selection that considers whole genome sequence and associated single-nucleotide polymorphisms (SNPs), rather than specific individual loci [20].

Understanding the genetic basis of breed differentiation through natural and artificial selection, production related traits, and disease will be greatly advanced by the availability of the genomic sequence of cattle. The foundation of the genomic sequence is provided by a clone-based physical map. Development of a clone-based physical map also offers a resource to accelerate discovery of polymorphisms within and between breeds, including causal polymorphisms contributing to a wide variety of bovine traits. The physical map is a source of genomic clones for sequencing templates and functional studies, and can also be employed to assist in the assembly of whole genome shotgun sequence [21-23].

The value of a clone-based map can be enhanced by establishing connections to the annotated genome sequences of closely related species [24], as well as to sequence tagged site (STS) maps of the same species. Links established between clones and annotated sequence can be used to identify specific clones containing genes of interest, and connections to genetic STS maps can indicate clones harboring QTL. Existing bovine genetic maps, however, have limited utility for identifying clones containing positional candidate genes near QTL, due to a lack of gene-specific genetic markers and a lack of recombination to separate closely linked markers within the existing bovine genetic maps [25-27]. Available gene-rich radiation hybrid (RH) maps [28-30] have greater short-range resolution than genetic maps, but their whole-chromosome ordering may be unreliable [31], and RH maps often lack many of the polymorphic markers that are needed to refine the locations of QTL. Exploiting the complementary resolution of genetic and RH data, a composite map can consolidate marker information to more efficiently indicate genes and sequence in the vicinity of QTL. Connections between a clone-based physical map, composite marker map, and annotated genome sequences will greatly facilitate the annotation of newly generated and assembled sequence. We report here the generation of a fingerprinted bacterial artificial chromosome (BAC)-based physical map, representing approximately 15.5-fold coverage of the bovine genome; the construction of a composite marker map from two linkage and three RH data sets; and connections with annotated human sequence and the largely unannotated draft bovine genome sequence.

## Results

### BAC clone fingerprinting

Clones from three BAC libraries, representing DNA from both beef and dairy cattle, were fingerprinted: 200,064 CHORI-240 [32], 94,848 RPCI-42 [32,33], and 44,948 TAMBT [34]. These include 18,982 CHORI-240 clones previously mapped on a low-coverage BAC fingerprint map [35], and 755 TAMBT clones selected for presence of a bovine marker. Fingerprints were attempted for 339,840 clones, and fingerprints for 290,797 clones (85.6%) were used to assemble the fingerprint map. These fingerprinted clones represent approximately a 15.5-fold coverage of the estimated 3.1 Gb bovine genome (Table 1).

**Table 1:** BAC library fingerprinting summary

Library	Library indicator*	Library construction enzyme^†^	Source DNA	Clones fingerprinted	Successful fingerprints	Clones in FPC^‡^	Average size (kb)^§^	Clone depth
CHORI240	E	*Mbo*I	Hereford bull L1 Domino 99373	200,064	170,644	169,283	169	9.5X
RPCI42	H	*Eco*RI/*Eco*RI methylase	Holstein bull	94,848	83,627	81,437	171	4.6X
TAMBT	T	*Hind*III	Angus bull, Angus cow	44,928	40,380	40,077	106	1.4X

Total				339,840	294,651	290,797	161	15.5X

*The one-letter prefix used in clone names to indicate the library source. ^†^All libraries were constructed from partially digested DNA. ^‡^After filtering. ^§^Based on the sum of the sizes of the *Hin*dIII fingerprint fragments identified for each clone, excluding vector-specific fragments.

A small subset of the BAC clones (approximately 5% of all fingerprinted clones) generated low complexity restriction digest fingerprint patterns, containing a single large molecular weight *Hin*dIII fragment (> 30 kb, the largest marker fragment), in addition to the expected vector-specific *Hin*dIII fragment(s). These clones appeared to lack *Hin*dIII sites within the insert, and were therefore unsuitable for fingerprint pattern-based assembly. Digestion of a small number of these clones with *Eco*RI generated fingerprints of one to four restriction fragments, each present in multiple copies (data not shown), suggesting that the inserts in these BAC clones were probably derived from regions of repetitive sequence [36]. Thus, while represented within the BAC library these genomic regions are under-represented within the fingerprint map.

### Aligning bovine BAC clones to the human genome

Following strategies used to increase contiguity, order and orient the mouse [24] and rat [21] BAC maps, bovine BAC end sequence (BES) reads were aligned with the reference human genome sequence [37,38]. At least one end read was available for 186,872 (64%) of the mapped BAC clones, and 149,865 (52%) had sequence data for both ends (Table 2, with additional data on their properties in Additional data file 1). A set of 12,273 paired BES alignments was obtained after filtering on alignment score, relative position and orientation. A single end sequence of 40,134 clones had a suitable match. In total, 48,325 clones in 480 contigs, along with 4,082 singletons, had sequence anchors to the human genome.

**Table 2:** Summary of BAC clones, sequences, and matches with composite map markers

	CHORI-240	RPCI-42	TAMBT	All
FPC map	169,283	81,437	40,077	290,797
Clone sequences*				
Single BES	26,900	4,472	5,513	36,885
Paired BES	121,205	20,173	8,487	149,865
Other	109	13		122
All	148,214	24,658	14,000	186,872
% Clones with sequence	87.6	30.3	34.9	64.3
Match marker^†^	21,077	3,770	744	25,591

*Clones with sequence deposited in GenBank genome survey sequence division. Single BES: sequence available for only one clone end. Paired BES: sequence available for both clone ends. Other: BAC clones with plasmid sub-clone sequences in addition to end sequences. ^†^Clone and marker sequences align, or clone and marker sequences match same Btau 3.1 whole-genome shotgun sequence contig [GenBank:AAFC03000000].

### BAC map assembly

Clone fingerprints were assembled into the International Bovine BAC Consortium (IBBMC) map, which consists of 655 contigs containing 257,914 clones, and 32,883 singletons (Table 3) [39-42]. This map is the result of first assembling an initial, high-stringency map, followed by merging based on similarity between clones at contig ends, number of unmatched restriction fragments at potential merge points, and comparative BES alignments. The initial 13,426 contigs and 34,189 singletons were assembled with FPC [43,44], before clone order within contigs was refined using CORAL[45].

**Table 3:** Fingerprint map summary

Number of contigs	655
Clones in contigs (% of total)	257,914 (89%)
Singletons	32,883
Average number of clones per contig	394
Largest number of clones in a contig	6,516
Contigs assigned to bovine chromosomes	397
Autosome and X assignment by comparative alignments	379
Y by SRY-positive probes	18
Number clones in assigned contigs (% of total)	252,971 (87%)
Average number of clones per assigned contig	637
Clones per autosome and X contig	663
Clones per Y contig	92
N50 size of assigned contigs	17.1 Mb
Average number of clones per unassigned contig	19
N50 size of unassigned contigs	0.5 Mb
Average number of contigs per autosome	10

When merging contigs, Sulston fingerprint similarity scores [46] were relaxed from the initial stringency, considering that the search space was limited to contig ends, and comparative alignments provided supporting evidence. A maximum of four unmatched restriction fragments across the merge point (fragments present in only one of the two merged contigs) allowed for: first, minor errors in fragment identification by BandLeader, the automated band calling software; second, the fact that *Hin*dIII does not cleanly excise the insert from the vector in the CHORI-240 and RPCI-42 BAC clones, resulting in two vector-insert junction fragments of unpredictable size that are not expected to be shared by neighboring clones; and third, the potential presence of polymorphic restriction fragments produced by indels, duplications, SNPs creating or destroying *Hin*dIII restriction sites, and other haplotype and/or breed-specific DNA variations that result in restriction fragment length differences. Automated scripts were employed throughout the merging process, although merges based on comparative alignments were manually inspected. Discrepancies between FPC/CORAL and human-based clone orders were identified, and manually evaluated in conjunction with the fingerprint images. Clones were rearranged to be consistent with human order only when the rearrangements were supported by fingerprint data.

### Assigning contigs to bovine chromosomes using comparative mapping data

A cattle-human comparative map [47] in combination with BES alignments to the human genome was used to assign, order and orient contigs on bovine chromosomes. Contigs were renumbered to reflect their chromosome assignments and relative order. A total of 379 of the 655 contigs were mapped onto bovine chromosomes by this process, 300 to autosomes (Table 3; Additional data file 2) and 79 to the X chromosome. An additional 18 contigs were tentatively assigned to the Y chromosome, five based on positive probes for the sex-determining region Y (SRY) marker, and the remainder based on fingerprint similarity to the SRY-positive contigs. The contigs assigned to autosomes had a mean size of 9.8 Mbp, based on the fingerprint data, and a mean of 813 clones per contig. Contigs assigned to the X chromosome were substantially smaller, with means of 1.7 Mbp and 92 clones per contig. Y-assigned contigs also averaged 92 clones per contig. Using this information, the genome size, including the contigs assigned to autosomes and X chromosome, was estimated to be 3.1 Gbp (Table 4). Chromosome assignments could not be made for 258 contigs, which lacked both human alignments needed for comparative assignment or marker-positive clones. These were relatively small contigs, containing an average of 19 clones, and had a mean size of 0.4 Mbp.

**Table 4:** Summary of map contig coverage of bovine chromosomes

Chromosome	No. of contigs	Estimated size (Mbp)*	Genome assembly size (Mbp)	Syntenic human chromosomes	Human genome coverage (Mbp)^†^
1	16	186	146	3, 21	155
2	10	160	125	1,2,15	135
3	11	145	116	1,2	118
4	12	141	110	7	119
5	18	143	118	12,22	115
6	19	135	111	4	117
7	14	135	100	1,5,19	105
8	11	129	103	4,8,9	106
9	15	128	95	6	108
10	8	121	95	5,14,15	98
11	16	123	101	2,9	113
12	15	108	77	13	91
13	8	104	83	10,20	91
14	11	97	82	8	96
15	13	101	75	11	75
16	11	98	72	1	76
17	8	92	70	4,12,22	81
18	11	82	62	16,19	70
19	12	78	63	17	70
20	5	83	68	5	75
21	7	80	63	14,15	63
22	2	73	59	3	63
23	10	62	48	6	56
24	12	73	60	18	71
25	10	54	42	7,16	49
26	5	58	47	10	51
27	2	48	43	4,8	41
28	3	52	40	1,10	44
29	5	61	45	11	44
X	79	138	99	X	119

Total	379	3088	2434		2616

*Size estimated from clone fingerprint data. ^†^Based on span of BES alignments to the reference human genome sequence.

### A composite bovine marker map

A 17,254-marker genome map of the 29 bovine autosomes and X chromosome (Additional data file 3) [40,48,49] was constructed from a composite of two linkage and three RH data sets. Marker data included those used to construct the Shirakawa Institute-US Department of Agriculture (SIAG-USDA) [26,27] and Alberta-Missouri (UAMU) [50] linkage maps, and the third generation Illinois-Texas (ILTX-2005) [28], Shirakawa Institute (SIAG) [29], and BovGen RH maps. For this work, the BovGen data set includes markers on the BovGen map [30], bovine sequencing project SNP [50], and other markers scored on the ComRad [51] panel. Strategies to exploit complimentary resolution characteristics of linkage and RH data [52,53] were employed to overcome inconsistencies between the individual maps. Sequence-based matching identified 17,254 unique markers from a total of 25,582 markers in the combined data sets, with 6,716 shared by at least two data sets and 6,173 common to at least one linkage and one RH data set (Table 5).

**Table 5:** Markers contributed by linkage and radiation hybrid data sets to the composite bovine map*

	BovGen	ILTX-2005	SIAG	SIAG-USDA	UAMU
BovGen	9,190	513	1,351	1,476	2,564
ILTX-2005		3,434	520	402	51
SIAG			5,513	3,218	48
SIAG-USDA				4,881	41
UAMU					2,564

Diagonal counts are markers contributed by the individual map; off-diagonals are counts of markers common to the two maps. There were 10,538 markers that appeared on only one map, 5,346 that appeared on two maps, 1,130 that appeared on three maps, and 240 that appeared on four or five maps. *BovGen: second generation [31] plus additional markers, including bovine sequencing project SNP [49] scored on 3,000 rad ComRad RH panel. ILTX-2005: markers from third generation map [29] scored on 5,000 rad Illinois/Texas panel. SIAG: microsatellite and EST markers scored on Shirakawa Institute 7,000 rad panel [30]. SIAG-USDA: genotypes and pedigree from Shirakawa-USDA linkage map [28]. UAMU: SNP genotypes and pedigree from eight-breed population [49].

The markers were mapped as 15,627 discrete entities, accounting for RH markers showing identical retention patterns within a panel. Three maps were computed for each chromosome. Only linkage and RH data were considered to determine an unassisted order. Ordering information from the BAC map and Btau3.1 draft assembly [54] was introduced for BAC- and sequence-assisted maps. Centimorgan (cM) and kilobase pair locations from the most likely of the three maps were interpolated using location database software [55], and confidence intervals were estimated. Mean separation between projected marker positions was 0.27 cM, or 228 Kbp. Estimated confidence intervals (CIs) indicate that 5,241 markers, occupying 4,639 positions, could not be repositioned relative to other markers without reducing likelihood. Expressed in cM, the median estimated CI is 0.8 cM, and 62.5% of markers have CI ≤ 1 cM. A tiny fraction of markers (0.1%) have extremely ambiguous positions with estimated CI ≥ 25 cM. Most of the extremely ambiguous markers are expressed sequence tag (EST)-based SNPs and have ambiguous placement on the SIAG-USDA linkage map [27].

### Marker-clone, marker-sequence and clone-sequence alignments

In addition to markers derived from BES, direct alignments between marker and clone-based sequences and indirect alignments using alignments of marker and clone sequences to whole genome shotgun sequence (WGS) contigs were used to anchor markers to the BAC fingerprint map. Matches between 10,313 markers and 25,591 BAC clones in 426 contigs were identified using e-PCR [56] and BLAT [57]. This includes 9,916 markers that matched 23,724 clones in 359 contigs assigned to autosomes or the X chromosome. Composite and BAC map chromosome assignments were consistent for 97.6% of the 8,902 markers that matched clones from a single chromosome. For 97.2% of the 1,014 markers that matched clones from multiple chromosomes (autosomes and X), at least one of the matched clones was assigned to the same chromosome as the marker. Additionally, clone-marker alignments suggested placement of 23 contigs that were not assigned to chromosomes by comparative alignments.

The comparative map-based chromosome assignments were supported by marker-clone alignments for 290 of the 300 contigs assigned to autosomes, and 66 of the 79 contigs assigned to X. The composite map indicated different chromosome assignments for only three autosome-assigned contigs (ctg7970 - BTA10; ctg17005 - BTA6; ctg25050 - BTA4). Each of these is located near a bovine-human breakpoint, so ambiguity in the bovine-human comparative map may have resulted in the apparent misassignment. For 112 of the 121 contigs containing clones that were assayed for a marker, comparative chromosome assignments agree with the physical probe assignments.

Further support for the comparative map-based chromosome assignments was provided by the first-generation bovine physical map produced by the French National Institute for Agricultural Research (INRA) [35]. The IBBMC and INRA BAC maps share 18,980 CHORI-240 clones, which allow 227 of IBBMC contigs to be joined with 653 INRA contigs. Chromosome assignments concur for 192 of the 214 assigned IBBMC contigs containing a shared CHORI-240 clone. Markers associated with INRA clones suggest chromosome assignments for the 13 unassigned IBBMC contigs containing a shared clone, although the assignments for two of these contigs remain ambiguous.

Strong agreement between composite and BAC map orders is indicated by Spearman's (ρ) and Kendall's (τ) rank correlation coefficients. Spearman's ρ, used to measure strength of the relationship between marker order along the two maps, ranges from 0.97 to 1.00 among autosomal markers matched to BAC clones. Coefficients are > 0.99 for 21 of the 29 autosomes. Kendall's τ indicates rearrangement necessary to reconcile orders, and ranges from 0.92 to 0.99 for the autosomal maps. Agreement between orders along the X chromosome is slightly weaker, with ρ = 0.91 and τ = 0.72. For all chromosomes, ρ exceeds τ, suggesting any rearrangements between the maps are predominately local, involving markers in close proximity to each other.

Composite map markers were also matched to the Btau3.1 draft assembly using e-PCR and BLAT. The draft assembly consists of contig sequences and intermediate scaffolds assembled with ATLAS [58], which were arranged on whole-chromosome scaffolds according to a set of bovine markers. A total of 15,746 markers matched 14,952 contig sequences, including15,498 markers matching whole-chromosome scaffolds, and 706 markers matching unassigned scaffolds. Further, 3,897 markers matched multiple contigs; for 3,495 of these, all contigs were within the same intermediate scaffold, 175 matched multiple intermediate scaffolds assigned to the same chromosome, and 227 matched scaffolds assigned to different chromosomes. Composite map and draft assembly chromosome assignments were consistent for 96.4% of the markers matching a single chromosome, and one assembly assignment concurred with the composite map for 92.7% of markers matching multiple chromosomes. Markers matched 668 unassigned scaffolds; 542 unassigned scaffolds matched a single marker, 97 matched more than one marker from the same chromosome, and 29 unassigned scaffolds matched markers from two or more chromosomes. Rank correlations suggest somewhat greater rearrangement between the composite map and draft assembly than was observed between the composite and BAC maps; ρ ranged from 0.89 to 1.00, with ρ of 13 chromosomes greater than 0.99; and τ was between 0.87 and 0.96. The level of agreement for the X chromosome was similar to the autosomes, with ρ = 0.98 and τ = 0.95.

End and other partial sequences from 156,783 fingerprinted BAC clones were also aligned to the assembled draft genome sequence. These include: 128,774 autosome- or X-assigned clones matched to whole-chromosome scaffold sequences; 26,587 autosome- or X-assigned clones matched to unassigned scaffolds; and 14,586 singleton BAC clones or clones in unassigned fingerprint contigs matched to whole-chromosome scaffold sequence. BAC map and draft assembly chromosome assignments were consistent for 96.1% of the aligned clones. All 379 autosome- and X-assigned fingerprint contigs contained at least one clone with sequence matching the bovine assembly. All autosomal BAC contigs contained at least one clone matching sequence assembled for that autosome, and the most frequently matched chromosome was consistent with BAC map chromosome assignment for 98.7% of the 300 autosome-assigned BAC contigs. Thirty-seven contigs exclusively matched sequence assembled for the assigned chromosome. Agreement between BAC map and assembly orders was less than that observed between the composite map and either the BAC map or assembly, with ρ between 0.88 and 0.95, and τ between 0.81 and 0.91 for the autosomes; for X, ρ = 0.79 and τ = 0.62.

After removing markers that matched multiple chromosomes, or had widely separated matches on a single chromosome, a set of 14,123 markers was identified to evaluate the likelihood of marker order along the Btau3.1 draft assembly, and to provide the starting point for sequence-assisted reordering of the composite map. Similarly, 7,780 markers with consistent BAC and composite map chromosome assignments matched to single or close clones on the BAC map were used to evaluate the likelihood of markers in the BAC map order, and initiate a BAC-assisted reordering of the composite map. Log_10_-likelihoods of markers ordered according to the assembly are lower than for the same markers in the unassisted composite map order (Additional data file 4). Differences in log_10_-likelihoods between markers ordered according to the BAC or unassisted composite map are not as pronounced; the BAC map orders for BTA17 and BTA20 are more likely than the unassisted order. After rearranging and adding markers not included in the starting order, the BAC-assisted order was more likely than the unassisted order for 20 autosomes (Additional data file 5). Sequence-assisted orders were never the most likely computed order, but the log_10_-likelihoods are intermediate between the unassisted and BAC-assisted orders for ten chromosomes.

**Figure 1:**
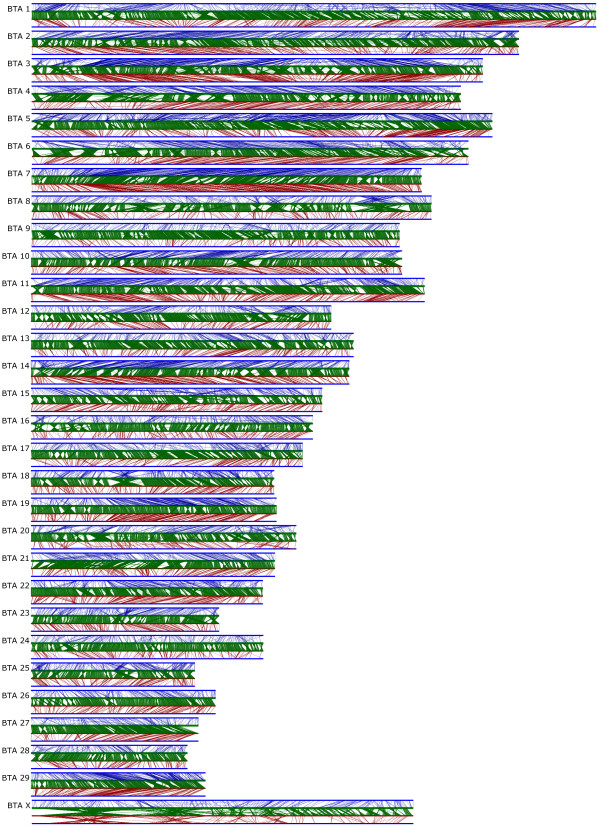
Comparison of the bovine BAC fingerprint map, composite marker map and Bt3.1 sequence assembly. For each chromosome, top and bottom lines are the composite map, the second line from the top is the assembly, and the third is the BAC map. The upper (blue) region depicts connections between the composite map to the assembly, the middle (green) connects the assembly and BAC map, and the lower (maroon) connects the BAC and composite maps.

The arrangement of markers and BAC clones along the composite map, BAC map and Btau3.1 assembly is depicted in Figure 1. Gbrowse [59] implementations to visualize the maps, including alignments to the BAC map, draft bovine and human sequence assemblies, and relative positions of QTL summarized from the literature [14,60] are available online [40,48,49].

## Discussion

A BAC physical map that spans the majority of the bovine genome has been constructed. Genome coverage by the BAC map is at least equal to that of the Btau 3.1 7X draft sequence assembly. Genome size estimated from the BAC map is 3.1 Gbp, somewhat larger than the 2.9 Gbp estimated by the bovine genome sequencing project, but at the low end of the 3.1 to 3.8 Gbp range of estimates obtained from different measures of haploid DNA content [61,62]. The use of three different BAC libraries each constructed using different restriction enzymes may have increased coverage over that possible with a single restriction enzyme, because certain genomic regions may not be clonable due to recognition site biases. Coverage by the BAC map may still be incomplete, however, because other highly repetitive, complex regions, such as telomeric and centromeric regions, may not be clonable with any enzyme, and may not be represented in any of the BAC libraries or the BAC map. Error in our estimates of genome size and genome coverage by individual contigs cannot be fully ascertained without a more finished assembly of bovine genomic sequence.

A composite linkage/RH map was also developed. This map consolidates available data to place markers in a consensus order and approximate positions in common cM and Kbp scales. The CarthaGene [31,51] procedures used to construct the bovine composite map have also been used to order markers on composite linkage maps of a parasitoid wasp [63], grapevine [64], rapeseed [65] and conifers [66], and a porcine map that combines linkage and RH data [67]. A related approach using weighted least squares is implemented in JoinMap [68] to compute composite linkage maps produced from different populations. Combining four linkage data sets for the *Picea mariana *× *Picea rubens *species complex [66] reported that similar maps were obtained with either JoinMap or CarthaGene.

**Figure 2:**
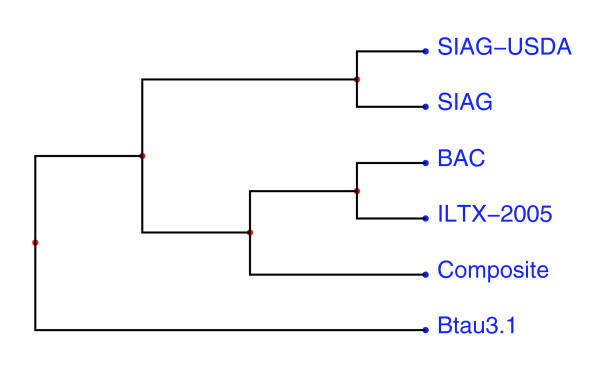
Phylogenic tree depicting relationships between whole-genome order of markers on bovine maps and sequence. Pairwise distances between maps are the 1 - τ, where τ is Kendall's rank correlation coefficient. Whole-genome τ values were computed by summing the number of inversions necessary to reconcile orders of each chromosome over all chromosomes. Maps included in the comparison are the Btau3.1 sequence assembly (Btau3.1), the BAC fingerprint map (BAC), the composite marker map, the third generation Illinois/Texas (ILTX-2005) radiation hybrid (RH) map [29], the Shirikawa (SIAG) RH map [30], and the SIAG-USDA linkage map [28]. BovGen RH and Alberta/Missouri (UAMU) linkage data also contribute to the composite map, but are not included here because an independent map of all markers scored on the BovGen panel is not available, and a lack of markers shared by UAMU and data sets other than BovGen precludes meaningful comparison.

There is significant global agreement among the bovine maps, although each of the contributing maps was rearranged to some extent relative to the composite map, and differences in order among the composite map, BAC map and sequence exist. Based on rearrangements among the well-connected whole-genome maps, the ILTX-2005 RH map is more similar to the composite and BAC map than either the SIAG RH or SIAG-USDA linkage map, and all the marker maps are more similar to the BAC map than to the Btau3.1 draft sequence assembly (Figure 2). Differences in log_10_-likelihoods suggest that the BAC map order is better supported by available linkage and RH data than the order of markers along the assembled sequence.

Examination of preliminary composite and BAC maps showed that discrepancies could not readily be resolved. Beyond the inevitable laboratory errors contributing to incorrect marker and clone order and contig membership, potential causes of discrepancies include sub-optimal orders of both maps, ambiguous orders, and error arising from spurious marker and clone sequence alignments. The composite map orders are probably less than optimal, because explicitly evaluating n!/2 possible orders, where n may represent a hundred or more markers on each chromosome, is computationally infeasible. The traveling salesman problem (TSP) approach [69,70] can be taken to implicitly determine optimal order for some RH data sets, but the combined data sets were not suitable for TSP analysis. Our approach of adding markers to an initial order, and using iterative *flips *and *polish *consistently produced the most likely order when testing alternative map construction strategies. The final order from this approach, however, is influenced by the starting order, and identifying the optimal order is not guaranteed. The process of computing maps from three different starting orders was intended to avoid bias towards any of the contributing marker maps, and to introduce fine-scale arrangements supported by external data that are not explicitly considered in the ordering process. Unassisted maps started from a pair of markers from opposite ends of each chromosome, to avoid bias towards any of the contributing maps. BAC-assisted orders started with markers ordered according to the BAC map, to introduce arrangements supported by fingerprint data. Similarly, sequence-assisted orders starting from markers ordered according to the draft assembly introduced assembly-based arrangements. This strategy of seeding the marker order according to the BAC map or assembly introduces marker arrangements that may not otherwise be evaluated. By considering these arrangements, the BAC map and sequence have some influence on the resulting composite map, but testing rearrangements of the seeded order ensures that the composite map will not include arrangements that are not supported by the marker data.

Some apparent discrepancies may result from ambiguously placed markers, which can occupy several positions on the composite map with equal likelihood. Ambiguity on the composite map also affects orders within regions between markers shared by two or more data sets, where there are no data to indicate the best merged order of markers that are unique to each data set. Other discrepancies may be attributed to spurious marker-clone alignments that appear only because of the erroneous match between a marker and clone, while some real discrepancies may be hidden by undetected links between markers and clones. Erroneous alignments may also contribute to errors on the composite map, if markers from different data sets are matched by incorrect alignments to the same GenBank bovine sequence or EST cluster (*Bos taurus *gene index or NCBI UniGene). The sequence based marker matching procedures were implemented to overcome inconsistent marker nomenclature, and result in identification of many more common markers than matches based solely on marker name. The sequence based procedures are dependent, however, on correct assembly of the sequences used to link markers. The two-point procedures to resolve inconsistent chromosome assignments will correctly break matches between markers that should be placed on different chromosomes, but will not detect incorrect matches between markers that should be separated on the same chromosome.

Repetitive sequence, particularly segmental duplications and other large scale genome variants [71], may exacerbate discrepancies between the marker and BAC maps. BAC clones that encompass or overlap a large duplication may be incorrectly assigned to the same contig, or correctly assigned but incorrectly ordered within a contig due to similarity of the fingerprints. Markers that match non-overlapping clones may indicate presence of repetitive sequence in the marker, clone and/or intermediate WGS sequence. Some rearrangements between the marker and BAC maps may be real, considering the diversity of genetic material represented by the maps, which include *Bos taurus *× *Bos indicus *crosses and several *Bos taurus *breeds. Breed-specific rearrangements have been shown in sheep [72], and structural variations, including inversions and inter- and intra-chromosomal translocations, have been detected among humans of diverse ethnic origin [73,74].

The level of disagreement between the composite map and assembled sequence can be reduced by rearranging the intermediate scaffold sequences according to the average (mean or median) composite map position of markers matching each scaffold, instead of ordering scaffolds according to minimum marker position within each scaffold, as was done for the current draft assembly. Basing the order of scaffolds on average rather than minimum marker position will be more accurate, especially if the minimum position is for a marker misplaced relative to other markers matching the scaffold. Remaining discrepancies between the composite map and assembly may have similar explanations as the inconsistencies between the composite and BAC maps: laboratory errors, ordering errors and ambiguous orders, spurious alignments, complications arising from repetitive DNA sequence, and structural variation among the genomes represented by the composite map and sequence.

Rearranging scaffolds to more accurately represent the marker map will also reduce disagreement between the assembled sequence and BAC map. Further refinement of the draft assembly may be achieved through greater consideration of the BAC map in the assembly process. Genomes represented by the BAC map and bovine reference sequence should be highly similar; reference DNA was obtained from a daughter of the bull used to construct the CHORI-240 library, which predominates the BAC map. The relationship coefficient (r_xy_) [75] of 0.954 between these two partially inbred individuals indicates their genomes will not be exactly the same, but will be much more similar than sire and progeny genomes resulting from mating unrelated parents (r_xy _= 0.5).

Besides the high density of alignments between WGS and BAC sequences, which can anchor and orient more genomic sequence than any marker map, paired BES alignments are especially valuable to orient and space adjacent sequence segments. Paired BES information has been used along with sequenced mammalian genomes to construct detailed framework maps [76]. In the current context, it can also be used as an independent check of the draft assembly and map coherency. Paired BES alignments [77] against the current draft assembly revealed possible clone identification and assembly errors. Systematic identification errors may affect BACs on 25 or more plates of the CHORI-240 library, containing approximately 3% of the BAC clones (A Ratnakumar and B Dalrymple, unpublished). Mis-identification may account for some chromosome assignment discrepancies between the composite and BAC map, and between the BAC map and draft assembly, but will not affect within-chromosome order comparisons, which do not include discrepant chromosome assignments. Considering estimated clone size, orientation and separation between paired BES alignments may improve the genome assembly. In a preliminary examination of a 50-scaffold region of BTA1, links between paired BES suggested a number of currently unassigned scaffolds should be placed in the region, and the neighbors and/or orientation of most scaffolds should change (B Dalrymple, W Barris and A Ratnakumar, unpublished). The discrepancies in order are predominantly local, consistent with the Kendall's correlation analysis.

Currently observed inconsistencies between the composite map, BAC map, and assembled sequence suggest that all may be improved to provide more accurate representations of the bovine genome. Some improvement of the composite map was realized by including fingerprint-scale information to reorder markers on the BAC-assisted map. Re-examination of the BAC map in light of the refined marker map may suggest further refinement of the BAC map, involving merges between contigs, splitting of contigs and other rearrangements. Similarly, examination of the draft sequence assembly relative to the BAC map may increase consistency with the BAC map and composite map. An iterative approach to refining the various maps is suggested. However, no rearrangement should occur for the sake of increasing consistency between the maps and sequence, unless appropriate data supporting the rearrangement are available. Discrepancies between the maps are relevant, pointing to regions where more experimental data are needed, where caution is required when examining contents of particular regions, and where bovine genomes may be prone to differ due to naturally occurring large-scale polymorphisms.

## Conclusion

The composite map consolidates available bovine mapping data, and leverages the complementary resolution of linkage and RH maps to provide a comprehensive marker map of the bovine genome. The BAC fingerprint map provides a resource to define comparative synteny, order and orient bovine genomic sequence, and estimate genome size and complexity. Further elucidation of the bovine genome is obtained from integration of the composite and BAC maps with annotated human sequence, draft bovine genomic sequence, and QTL describing genomic regions associated with phenotypic variation. QTL, described relative to the marker maps, can be anchored to underlying bovine genomic sequence through the BAC map. Annotation, transferred through the high-resolution bovine-human BAC-based comparative map, supplies information about genes and gene function needed to enhance our understanding of biological mechanisms affecting agriculturally important traits. The BAC map is a valuable resource for the development of genomic tools to further our knowledge of evolution of this species, which has undergone natural, undocumented and documented artificial selection, and which may contribute further insight into human conditions.

## Materials and methods

### BAC library resources

Clones from three BAC libraries were fingerprinted: CHORI-240 derived from Hereford bull L1 Domino 99375 DNA [32] (PJ de Jong, K Osoegawa and C Shu, unpublished) and RPCI-42 derived from Holstein bull DNA [32,33] were constructed at BACPAC Resources Centre [32]. The TAMBT library, containing clones derived from Angus bull and cow DNA, was constructed at Texas A&M University [34] (CA Gill and SL Davis, unpublished).

Based on recorded pedigree, the Hereford bull used for CHORI-240 has an inbreeding coefficient (F_x_) of 0.31 [75]. His daughter used for the bovine genome sequencing project has F_x _= 0.30, and the r_xy _between these two individuals is 0.954 (MD MacNeil, personal communication).

### BAC clone fingerprinting

Fingerprints were generated using an agarose-gel based methodology [23,78,79]. Briefly, BAC clones were cultured overnight in 96-well format and DNA was extracted using an alkaline lysis procedure. The BAC DNA was digested with *Hin*dIII (New England Biolabs, Ipswich, MA, USA) and the resulting fragment sizes were resolved by electrophoresis on agarose (Cambrex BioWhittaker, Walkersville, MD, USA) gels. Gels were stained after electrophoresis with SYBR Green I (Invitrogen, Carlsbad, CA, USA) and scanned using a Molecular Dynamics Fluorimager 595. The digitized images were lane tracked interactively using Image software [46,80,81] and restriction fragments were automatically identified and sized using BandLeader [82]. Restriction fragments within a size range of approximately 600 bp to 30 Kbp were collected. *Hin*dIII fragments predicted by their size as being derived from sequences internal to the vector were removed from the fragment list for each clone.

### BAC end sequencing

End sequencing of BAC clones from the three libraries was performed as part of the International Bovine BAC Mapping Consortium effort. End sequence reads have been deposited in GenBank [GenBank:BZ830806-BZ891831; BZ896446-BZ956676; CC447354-CC447937; CC466118-CC470858; CC470880-CC596504; CC761663-CC775995; CC902786-CC927336; CG917936-CG918393; CG976420-CG992944; CL526294-CL527670; CL603252-CL610093; CL864822-CL865757; CR792448-CR792448; CR792463-CR812463; CR812501-CR846076; CR846104-CR846104; CW848133-CW848163; CZ12846-CZ27312; CZ404298-CZ429751] (Table 6, Additional data file 1) and in the NCBI Trace Archive.

**Table 6:** Summary of CHORI-240, RPCI-42 and TAMBT BAC clones represented by sequences deposited in the genome survey sequence division of GenBank

Source*	All sequences^†^	IBBMC sequence^‡^	All clones	IBBMC clones
TIGR	61,023	61,023	29,286	29,286
UIUC	107,709	75,070	51,921	38,979
EMBRAPA	43,023	43,023	21,571	21,571
BCGSC	125,597	125,597	58,368	58,368
OU	26,863	25,486	14,844	14,000
BARC	26,982	25,454	10,862	10,857
USMARC	31,355		117	
				
Total	422,552	330,167	186,969	159,061

*TIGR, The Institute for Genomic Research [GenBank:BZ830806-BZ891831]; UIUC, University of Illinois at Urbana-Champaign [GenBank:BZ896446-BZ956676; CC447354-CC447937; CC761663-CC775995; CW848133-CW848163]; EMBRAPA, Embrapa Genetic Resources and Biotechnology [GenBank:CC466118-CC470858; CG917936-CG918393; CG976420-CG992944; CL603252-CL610093; CZ012846-CZ027312]; BCGSC, British Columbia Cancer Agency Genome Sciences Centre [GenBank:CC470880-CC596504]; OU, University of Oklahoma Advanced Center for Genome Technology [GenBank:CC902786-CC927336; CL526294-CL527670; CL864822-CL865757]; BARC, USDA-ARS-Beltsville Agricultural Research Center [GenBank:CZ404298-CZ429751]; USMARC, USDA-ARS-US Meat Animal Research Center. ^†^Includes CHORI-240 and RPCI-42 plasmid sub-clone sequences and end sequences not identified with the IBBMC effort. ^‡^International Bovine BAC Mapping Consortium. In addition, 53,556 ends of 26,936 clones from the INRA BAC library were sequenced in the IBBMC effort [GenBank:CR792448-CR792448; CR792463-CR812463; CR812501-CR846076; CR846104-CR846104].

### Anchoring BAC clones to the human genome assembly

BES were aligned to repeat-masked human genome sequence assemblies (UCSC hg17, based on NCBI Build 35; and UCSC hg18, based on NCBI Build 36.1) [38] using BLASTN [83] with options -z 3095016460 -m 8. Only the best achieved hits were considered. Paired-end alignments were required to satisfy the following criteria: E-value ≤ 1e-2 for both alignments, with alignments ≤ 400 Kbp apart and in opposite orientations. Single end alignments were required to have E-values ≤ 1e-8. As described below, alignments to hg17 were used in conjunction with the bovine-human comparative map described by [47] to merge contigs and arrange contigs on chromosomes. Alignments to hg18 were used to define the bovine BAC-human comparative map (Additional data file 2).

### Initial fingerprint map assembly

BAC fingerprints were assembled using FPC [43,44]. The initial assembly was performed using the default parameters (tolerance 7; min bands 3; best = 10; no CpM) and a cutoff value of 1 × 10^-16^. The fingerprinted clones were then screened and filtered as follows to remove fingerprint patterns indicative of artifactual data. First, a software application, MapMop, was developed to calculate the distributions of insert size and number of restriction fragments for the clones. Based on these distributions, filtering parameters were determined to identify 'outlier' clones that represented extremes for insert size and/or number of restriction fragments, suggesting problematic data such as that resulting from partial digestion of the BAC DNA, or the presence of DNA from more than a single BAC clone (cross-well contamination). Second, initial filtering parameters were assessed by visual inspection of representative clone fingerprints and were adjusted to minimize the retention of poor quality data while limiting the loss of high quality data. Filtering parameters were determined separately for individual libraries, for clones within contigs and for clones that were not assembled into contigs ('singletons').

Following filtering, the remaining fingerprints were again assembled at a cutoff value of 1 × 10^-16^. The DQer function in FPC was used to reassemble any contigs that contained 'Q' clones, which exhibit an unusual number of extra bands or gaps between bands matching the Q clone to other clones, resulting in false positive overlaps between putative contigs. The presence of Q clones within a contig is suggestive of misassembly due to false-positive fingerprint matches. The DQer function reassembled the clones within the contig with successively increasing stringency until no Q clones remained. The resulting contigs were subsequently processed with an automated clone ordering application, CORAL [45], to refine clone order within the contigs.

### Merging map contigs

Contigs were merged in multiple steps using automated scripts to identify and execute joins between contigs. Initial merges were performed using only the fingerprint data to identify candidate merge points for contigs that satisfied the criteria of Sulston scores of 9 × 10^-8 ^for at least two contig end clones, and that had no more than four unconfirmed fragments at the merge point. An unconfirmed fragment is one that is present in the fingerprint of the end clone of one contig but without a match to fragments in fingerprints of end clones in either of the contigs at the merge point. A subset of merged contigs was manually reviewed to evaluate whether these parameters resulted in incorrect merges.

After making the initial merges, BES alignments to the human sequence assembly were examined to identify candidate contig merges based on their human genome sequence coordinates. Merges were made in cases where the Sulston score match between end clones was 9 × 10^-7 ^and there were four or fewer unconfirmed fragments. Automated scripts and human inspection were used to identify and eliminate incorrect clone orders and merges at multiple points throughout the merging process.

### Estimating map contig size

Contig sizes were estimated from fingerprint data with an algorithm that compared the restriction fragments of overlapping clone pairs in the canonical clone set for each contig. Canonical clones are the set of non-redundant overlapping clones spanning a contig that each represent a unique complement of restriction fragments in their fingerprint, such that the remaining non-canonical clones within the contig are subsumed by the canonical clones (that is, all the restriction fragments in the fingerprint of a non-canonical clone are completely represented in one of the canonical clones). The unique fragments for each canonical clone were identified, and their sizes were summed to estimate the overall size of the contigs. Specifically, the algorithm performed the following for each contig: first, sum the sizes of all the fragments in the left-most canonical clone in the contig to create a cumulative size estimate; second, identify the next canonical clone immediately to the right and identify its unique fragments (any fragments not shared with the previous canonical clone to the left or the next canonical clone to the right), then add the sizes of these unique fragments to the cumulative size estimate; third, repeat step 2 until all unique fragments in the canonical clones have been identified and summed to give a total size estimate for the contig. Fragments were considered to be the same if their calculated standard mobilities were within ten mobility units of each other.

### Composite bovine map

The composite map was built using data from two independent linkage maps, as well as markers scored for presence or absence on three independent whole-genome RH panels (Table 5). Genotypes and pedigrees were those used for the SIAG-USDA linkage map [26] with EST-based SNP [27], and the UAMU SNP linkage map [50]. The two maps anchor 7,404 markers along 30 bovine chromosomes (29 autosomes and the X chromosome), albeit at relatively low resolution with many markers not separated by observed recombination. Markers scored on at least one of the three independent RH panels provided the basis to separate closely linked markers as well as place 9,850 additional markers that were not represented in the linkage maps. Radiation hybrid data included those used for the ILTX-2005 map scored on the 5,000 rad Illinois-Texas panel [28], the SIAG map scored on a 7,000 rad panel [29], and the BovGen map [30] with markers scored on the 3,000 rad ComRad panel [51]. For this work, additional markers scored on the ComRad panel are also considered part of the BovGen data set. These include Illumina BeadStation-scored SNP [50] identified from the bovine genome sequencing initiative, and a number of amplified fragment length polymorphism markers. The UAMU linkage map SNPs are a subset of the sequencing project SNPs scored on the ComRad RH panel.

**Figure 3:**
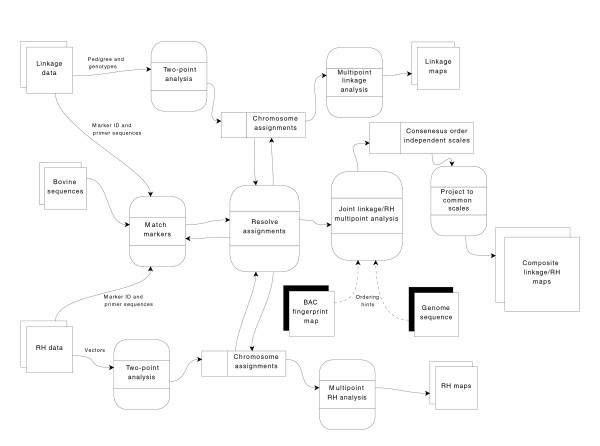
Data flow diagram of the composite map construction process.

Composite map construction processes are depicted in Figure 3. Markers shared by two or more data sets were identified, assigned to chromosomes, and each chromosome was ordered. Markers were matched across data sets using a combination of marker names, primer sequence, and primer sequence alignments to the same bovine sequence or EST cluster. Markers with identical primer sequences were considered to be the same, regardless of marker name. Additional matches between data sets were obtained using e-PCR [56] to align primer pairs with GenBank bovine and *Bos taurus *Gene Index sequences (BtGI, version 11) [84]. Primer pairs that matched the same sequence, with no more than one mismatch or gap, were considered to represent the same marker, as were markers matching different ESTs from the same *Bos taurus *UniGene (NCBI *Bos taurus *UniGene, build 68) [85]. Name-based matches were made only between those markers with identical names and no sequence or mapping evidence to the contrary (markers without primer sequence but sharing the same name and chromosome assignment were matched, and markers sharing the same name but having different primer sequences were not matched unless the different primers hit the same sequence). Matches were checked for consistency with original chromosome assignments from the independent maps. When markers assigned to different chromosomes in the independent maps were matched, data used to match markers, results of two-point analyses, and comparative human alignments were examined. Matches were preserved, and markers reassigned, when two-point linkage supported reassignment. Sequence- and name-based matches were ignored if two-point linkage supported the original assignments. Comparative alignments were used to break ties, when two-point LOD scores and distances for a marker indicated equally likely assignment to two or more chromosomes, and the comparative alignment supported assignment to one of those chromosomes.

Independent two-point analyses of each RH data set were conducted to identify markers to be included on the composite map. Markers common to each RH data set and the SIAG-USDA linkage data were identified. Starting from this set, markers linked with a two-point LOD > 7.0 and < 40 centiRad (cR) separation from previously linked marker were identified in successive passes through the two-point results. Markers assigned to each chromosome-specific linkage group were ordered by an automated process [53], seeded with three different orders. An initial, unassisted order started with a pair of markers from opposite ends of the chromosome. The BAC-assisted order started with the BAC map order of markers that were matched to BAC clones, and the sequence-assisted order started with the assembly order of markers that were anchored to the assembly. Only markers with consistent chromosome assignments, and unambiguous placement on the BAC map or sequence were included in the starting orders. Log_10_-likelihoods of markers in the BAC- and sequence-based starting orders were computed, iterative *polish *and *flips *procedures were applied to determine a more likely order of markers included in the starting order, remaining markers assigned to the chromosome were sequentially added, and the final order was determined with iterative *polish *and *flips*. Confidence intervals surrounding marker placement were approximated from *polish *applied to the final order. Cytogenetic band, cM and Kbp positions for each marker were computed with a modification of the *ldbf *program [55], using the native unit (cM and cR) maps in the most likely consensus order determined from the three different starting orders.

### Marker, clone and genomic sequence alignment

Markers placed on the composite map were associated with BAC clones in the fingerprint map via direct alignment of marker and BAC end- or sub-clone sequences, and indirect alignment of marker and BAC sequences to the same WGS contig sequence. Direct marker-clone, and indirect marker-WGS-clone alignments were identified by e-PCR and BLAT. Alignments of primer pairs against BAC clone and WGS by e-PCR allowed no more than one mismatch or gap. BLAT was used to align marker sequences with BES and WGS contigs, and to align BAC clones with WGS contigs. BLAT alignments required exact matches and a bit score of at least 100, 99.5% identity with a score of 250, or 99% identity and a score of 500. Direct matches were identified from markers matching BAC clone sequences, and indirect matches from markers and BAC clones matching the same WGS contig.

Marker sequence used for BLAT alignments was obtained from GenBank when information to associate the marker with a GenBank accession number was available, or from STS sequence supplied with the marker. When only primer sequences were available, the e-PCR results used to match markers across data sets were also used to construct an amplimer sequence. Sequence between primer locations was extracted from each e-PCR match, and assembled with Phrap (version 0.990329) [86]. This assembled sequence was used to represent the marker only when the Phrap assembly resulted in a single contig.

The collection of GenBank bovine sequence used for e-PCR alignments to match markers across linkage and RH data sets includes bovine sequences deposited in the genome survey sequence (GSS), STS, patent (PAT), mammalian (MAM), and EST divisions of GenBank after release of the second bovine draft assembly. Bovine sequences were identified by taxonomy identification numbers for *Bos taurus *(txid 9913), *Bos indicus *× *Bos taurus *(txid 30522), *Bos taurus *× *Bos indicus *(txid 30523), and *Bos *sp. (txid 29061, associated with some patent [PAT] division sequences). The MAM division sequences included WGS contig sequences from the second bovine sequence assembly [GenBank:AAFC02000000]. Markers and BAC sequences were also aligned with WGS contigs from the third bovine draft assembly [GenBank:AAFC03000000] and scaffold sequences (whole-chromosome scaffolds [GenBank:CM000177-CM000206]; unassigned scaffolds [GenBank:CH974204-CH980624]).

### Alignment of the BAC fingerprint and composite marker maps

#### Discrepancy resolution

Associations between markers and clones were used to assess and refine chromosome assignments and the order of fingerprint contigs, where the contigs were initially assigned and ordered along chromosomes using end-sequence alignments to human sequence and bovine-human comparative maps [29,47]. For each fingerprint contig containing clones associated with markers mapped on a preliminary composite map, marker positions unambiguously linked to a single contig were identified, and uninterrupted runs of marker positions associated with a single contig were determined. Marker positions were regressed on corresponding clone position within a contig for the run with the greatest number of markers to predict clone position on the composite map. Predicted positions of clones within contigs linked to the composite map by a single marker position were set to that marker position. Discrepancies between predicted clone positions, and composite map positions of markers linked to those clones were identified. Fingerprints of clones linked to the observed discrepancies were examined, and clones were rearranged to eliminate discrepancies only if the rearrangement was supported by fingerprint data. The preliminary composite map used included SIAG-USDA linkage data, and RH markers and vectors from SIAG, the second generation Illinois-Texas map [47], and a subset of the BovGen data used for the current map.

#### BAC-assisted composite map

Direct marker-clone and indirect marker-WGS-clone alignments were used to identify the set of markers from the current composite map with consistent, unambiguous alignments to the BAC map. The order of these markers on the BAC map was used to seed the ordering process for each chromosome. A map of the markers matching the BAC map was computed, followed by iterative *polish *and *flips *to reorder markers in that set. Markers assigned to the chromosome, but not matching BAC clones, were added with *buildfw*, and a final BAC-assisted order obtained from iterative *polish *and *flips *after all assigned markers were included.

## Additional data files

The following additional data are available with the online version of this paper. Additional data file 1 describes analyses of BAC end sequences. Additional data file 2 contains figures showing the bovine-human comparative map defined by the BAC map and BES alignments. Additional data file 3 is a table containing placement of markers on the composite map. Additional data file 4 is a table of log_10 _likelihoods of markers ordered according to the BAC map, Btau3.1 draft assembly, and unassisted composite map. Additional data file 5 is a table of log_10 _likelihoods for the unassisted, BAC-assisted and sequence-assisted composite maps.

## Abbreviations

BAC, bacterial artificial chromosome; BES, BAC end sequence; CI, confidence interval; cM, centimorgan; cR, centiRay; EST, expressed sequence tag; IBBMC, International Bovine BAC Mapping Consortium; ILTX, Illinois-Texas; INRA, French National Institute for Agricultural Research; QTL, quantitative trait loci; RH, radiation hybrid; SIAG, Shirakawa Institute; SNP, single-nucleotide polymorphism; SRY, sex-determining region Y; STS, sequence tagged site; UAMU, Alberta-Missouri; USDA, US Department of Agriculture; WGS, whole genome shotgun sequence.

## Authors' contributions

The first three authors contributed equally to the work. RC and JES developed the BAC fingerprint and BAC-human comparative maps. WMS and MH developed the composite map and integrated marker maps with the BAC map and draft assembly. CAA, CAG, AEvdW, DML, HAL, SSM, SMcK, BM, MG and LS developed probes and screened BAC clones. RB, RC, DML and WMS analyzed BES. IEB, RH, SJMJ, MAM, CAM, NHY and GY were involved in fingerprinting, BAC end sequencing and BAC map creation. SY, CPVT, TSS, ARC, MMC, DML, AE, HAL, RT, LCS, LKM, AR, and SZ were involved in BAC end sequencing, coordinated by HAL and JES. GLB, JWK, SMK, TPLS, WMS, JFT, and RDS contributed to linkage maps. DML, AEvdW, HAL, JEW, JLW, JA, OJ, SMcK, SSM, BM, AE, SF, MG, MB, AR and LS were involved in RH map development. LS, AE, MB, SF, and MG contributed to INRA BAC map and integration of IBBMC and INRA maps. PdJ, KO, and CAG participated in BAC library development. DLA, BPD, HS and WMS contributed to the gbrowse web sites. AE, JLW, FWN, and JWK conceived the composite map. TPLS, JCM, SMK, RDG, HAL and JEW were involved in conceiving and planning the project. WMS, JES, RC, TPLS and RB drafted portions of the manuscript. MH, JFT, RT, JCM, DLA, ARC, AMC, BPD, AE, CAG, JWK, DML, MAM, TSS, CPVT, and JLW edited the manuscript. All authors read and approved the final manuscript.

## Supplementary Material

Additional data file 1 Analyses of BAC end sequences. Click here for file

Additional data file 2 Figures showing the bovine-human comparative map defined by the BAC map and BES alignments. Click here for file

Additional data file 3 Placement of markers on the composite map. Click here for file

Additional data file 4 Log_10 _likelihoods of markers ordered according to the BAC map, Btau3.1 draft assembly, and unassisted composite map. Click here for file

Additional data file 5 Log_10 _likelihoods for the unassisted, BAC-assisted and sequence-assisted composite maps. Click here for file
