# Supplementary material for: A physical map of the bovine genome
Source: Genome Biol. 2007 Aug 14;8(8):R165. doi: 10.1186/gb-2007-8-8-r165 (PMC2374996; doi:10.1186/gb-2007-8-8-r165)
Supplement: Additional data file 1 — Analyses of BAC end sequences. [file gb-2007-8-8-r165-S1.doc]

**Supplementary data**

The following tables provide more detail about the properties of the 366,659 BAC end sequences used in the construction of the physical map of the bovine genome.

**Sequence read numbers**
**Table 1**. The number of sequences by sequencing centre, abbreviations as per NCBI*

| **Centre** | **Number of sequences** |
| --- | --- |
| BARC | 20,730 |
| BCGSC | 112,076 |
| EMBRAPA | 37,136 |
| OU-N | 23,635 |
| TIGR | 53,789 |
| UIUC | 92,580 |
| USMARC | 26,687 |

*26 sequences lacked centre information.

**Table 2**. Sequences per library

| **Library** | **Number of sequences** |
| --- | --- |
| CHORI-240 | 296,599 |
| RPCI-42 | 47,573 |
| TAMBT | 22,487 |

**Contamination**

The sequences were filtered for contamination, and sequence quality using Seqclean (<http://www.tigr.org/tdb/tgi/software/seqclean_README> ). A default length cutoff of 100bp was used and the UniVec library as of 09/2006. This resulted in 7,811 sequences (2.1%) having fewer than 100bp of good quality sequence. Only 354 were removed due to vector or E. coli contamination. 77,300 sequences were trimmed for a variety of reasons.

**Sequence properties**

***Sequence length***

The total length of all sequences was 221,115,378 bp

**Table 3.** Number and length of sequences by sequencing centre

| **Centre** | **Number of sequences** | **Total length of sequences** |
| --- | --- | --- |
| BARC | 20,730 | 12,055,270 |
| BCGSC | 112,076 | 79,251,852 |
| EMBRAPA | 37,136 | 14,910,344 |
| OU-N | 23,635 | 18,524,044 |
| TIGR | 53,789 | 28,866,911 |
| UIUC | 92,580 | 49,960,942 |
| unknown | 26 | 15,012 |
| USMARC | 26,687 | 17,531,003 |

**Table 4.** Number and length of sequences by library

| **Library** | **Number of sequences** | **Total length of sequences** |
| --- | --- | --- |
| CHORI-240 | 296,599 | 174,805,196 |
| RPCI-42 | 47,573 | 28,689,174 |
| TAMBT | 22,487 | 17,621,008 |

***Paired end reads***

These reads consist of a subset of the above sequences. They come from 3 BAC libraries and in the following context a “paired clone” is BAC clone with 2 end sequences and an unpaired clone is a BAC clone with end sequence.

**Table 5.** Sequences per cloneID by library*

|  | **Number of clones with** | | |
| --- | --- | --- | --- |
| **Library** | **One sequence** | **Two sequences** | **More than two sequences** |
| CHORI-240 | 26,900 | 119,920 | 1,394 |
| RPCI-42 | 4,472 | 20,170 | 16 |
| TAMBT | 5,513 | 8,487 | 0 |

* Some clones were sequenced more than once, unpaired are defined as those with only one sequence

**Table 6**. Paired and total clone sequences and percentage of unpaired reads by library

| **Library** | **Paired clones** | **Unpaired clones** | **Total clones** | **Percent unpaired clones / total clones [%]** |
| --- | --- | --- | --- | --- |
| CHORI-240 | 121,314 | 26,900 | 148,214 | 18.15 |
| RPCI-42 | 20,186 | 4,472 | 24,658 | 18.14 |
| TAMBT | 8,487 | 5,513 | 14,000 | 39.38 |

**Table 7.** Paired and total clone sequences and percentage of unpaired reads by sequencing centre

| **Centre** | **Paired clones** | **Unpaired clones** | **Total clones** | **Percent unpaired clones / total clones [%]** |
| --- | --- | --- | --- | --- |
| unknown | 6 | 14 | 20 | 70.00 |
| USMARC* | 117 | 0 | 117 | 0.00 |
| BARC | 8,502 | 2,360 | 10,862 | 21.73 |
| OU-N | 8,791 | 6,053 | 14,844 | 40.78 |
| BCGSC | 53,708 | 4,660 | 58,368 | 7.98 |
| UIUC | 40,659 | 11,262 | 51,921 | 21.69 |
| TIGR | 22,718 | 6,568 | 29,286 | 22.43 |
| EMBRAPA | 15,565 | 6,006 | 21,571 | 27.84 |

*These sequences include internal BAC clone reads.

***Repetitive sequence***

Repeatmasker was used with standard settings and Repbase version : 11.12 (January 2007) for *bos taurus*. The results were:

- 266,977 sequences (72.8 % of all sequences) were partially or fully masked.
- 172,866 of masked sequences have unmasked stretches longer than 100 bp.
- Total unmasked sequence amounts to 74,867,468 bp (33.9 %).
- 266,977 masked sequences contain 146,247,910 bp of masked sequence (i.e. 548 bp per masked sequence).

**Table 8.** Number of masked (>100bp unmasked) and unmasked sequences and total by sequencing centre

| **Centre** | **Number of masked seqs with > 100 bp unmasked** | **Number of unmasked seqs** | **Total number of seqs with > 100 bp unmasked (percent of total seqs per sequencing centre)** |
| --- | --- | --- | --- |
| BARC | 11,019 | 5,245 | 16,264 (78.46%) |
| BCGSC | 60,857 | 22,032 | 82,889 (73.96%) |
| EMBRAPA | 10,661 | 14,414 | 25,075 (67.52%) |
| OU-N | 14,269 | 7,230 | 21,499 (90.96%) |
| TIGR | 22,379 | 14,893 | 37,272 (69.29%) |
| UIUC | 42,185 | 24,018 | 66,203 (71.51%) |
| unknown | 11 | 10 | 21 (80.77%) |
| USMARC | 11,485 | 11,840 | 23,325 (87.40%) |

**Table 9.** Number of masked (>100bp unmasked) and unmasked sequences and total by BAC library

| **Library** | **Number of masked seqs with > 100 bp unmasked** | **Number of unmasked seqs** | **Total number of seqs with > 100 bp unmasked (percent of total seqs per library)** |
| --- | --- | --- | --- |
| CHORI-240 | 133,712 | 80,871 | 214,583 (72.35%) |
| RPCI-42 | 25,289 | 12,307 | 37,596 (79.03%) |
| TAMBT | 13,865 | 6,504 | 20,369 (90.58%) |

***Percentage that have a BLAST match against bovine refseqs (12/2006)***

Megablast was used with the following options

**-F "m D" -U T -D 2 -m 8**

The top hit, where present, was extracted and those with a percent identity above 95 and an E value below 0.01 retained. The results were:

- 18,831 sequences (5.14 % of all sequences) have a BLAST hit, 347,828 sequences don't. The average identity was 99.47% for the matching region.
- Out of 18,831 sequences 6,050 sequences (32.1 %) do have multiple BLAST hits.
- 16,028 (4.37 % of all sequences) do have a BLAST hit with percent identity > 95 and evalue < 0.01, 350,631 sequences don't.
- Out of the 16,028 hits 11,000 (68.6 %) are against “Predicted” rather than curated sequences.

In addition 67 refseq sequences had hits against the repeats database (Repbase version 11.12, January 2007). All of these 67 refseq sequences also have hits against the BAC end sequences with 104 matches in total.

***Matches against bovine 3.1 assembly for % hits and % homology***

Megablast was used to search the chromosomal sequences of the bovine 3.1 assembly for the sequences (lower case masked sequences as described previously) with following options

**-F "m D" -U T -D 2 -m 8**

The top hit, where present, was extracted and those with a percent identity above 95 and an E value below 0.01 retained. The results were:

- 274,261 sequences (74.80 %) do have a BLAST hit, 92,398 sequences don't. The average identity was 99.29 % for the matching region.
- Out of 274,261 sequences 71,935 sequences (26.2 %) do have multiple BLAST hits.
- 265,899 sequences (72.52 %) do have a BLAST hit with percent identity > 95 and evalue < 0.01, 100,760 sequences don’t.
- Note the 3.1 assembly does not have a Y chromosome and matches were only to sequences assigned to chromsomes.

**Table 10.** Summary of hits by chromosome

| **Accession number** | **Chromosome** | **Number of hits with percent identity > 95 and evalue < 0.01** |
| --- | --- | --- |
| CM000177 | 1 | 14,137 |
| CM000178 | 2 | 12,729 |
| CM000179 | 3 | 11,296 |
| CM000180 | 4 | 11,204 |
| CM000181 | 5 | 12,499 |
| CM000182 | 6 | 12,622 |
| CM000183 | 7 | 9,015 |
| CM000184 | 8 | 9,351 |
| CM000185 | 9 | 8,795 |
| CM000186 | 10 | 9,033 |
| CM000187 | 11 | 9,467 |
| CM000188 | 12 | 7,087 |
| CM000189 | 13 | 7,818 |
| CM000190 | 14 | 7,787 |
| CM000191 | 15 | 6,434 |
| CM000192 | 16 | 6,262 |
| CM000193 | 17 | 7,325 |
| CM000194 | 18 | 5,869 |
| CM000195 | 19 | 5,763 |
| CM000196 | 20 | 7,586 |
| CM000197 | 21 | 5,678 |
| CM000198 | 22 | 5,679 |
| CM000199 | 23 | 4,733 |
| CM000200 | 24 | 5,652 |
| CM000201 | 25 | 4,214 |
| CM000202 | 26 | 4,385 |
| CM000203 | 27 | 5,740 |
| CM000204 | 28 | 3,599 |
| CM000205 | 29 | 4,442 |
| CM000206 | X | 4,645 |

***Percentage and depth of hits per assembled chromosome***

The results shown in table 11 below provide very similar and uniform coverage of the assembled genome with the obvious exception of chromosome X because CHORI-240, RPCI-42 and TAMBT are all libraries of male genomes. Chromsomes 6 and 27 have a somewhat higher % of the chromosome covered and also depth of coverage suggesting more frequent restriction sites for the enzymes used to create these libraries.

**Table 11.** Percentage coverage and depth of coverage by chromosome for the 3.1 B Taurus assembly

| **Chromosome** | **Positions covered** | **Positions uncovered** | **Positions covered** | **Depth of coverage*** |
| --- | --- | --- | --- | --- |
| 1 | 3,733,608 | 142,466,247 | 2.55 % | 1.10 |
| 2 | 3,409,405 | 122,421,310 | 2.71 % | 1.10 |
| 3 | 3,021,714 | 113,465,556 | 2.59 % | 1.08 |
| 4 | 3,098,271 | 107,774,744 | 2.79 % | 1.09 |
| 5 | 3,185,694 | 115,805,516 | 2.68 % | 1.16 |
| 6 | 3,061,762 | 108,698,233 | 2.74 % | 1.26 |
| 7 | 2,447,538 | 98,396,333 | 2.43 % | 1.06 |
| 8 | 2,577,979 | 101,136,412 | 2.49 % | 1.06 |
| 9 | 2,522,754 | 92,507,665 | 2.65 % | 1.05 |
| 10 | 2,432,739 | 93,385,915 | 2.54 % | 1.07 |
| 11 | 2,569,252 | 99,065,806 | 2.53 % | 1.05 |
| 12 | 1,989,930 | 75,671,286 | 2.56 % | 1.04 |
| 13 | 2,138,146 | 81,233,337 | 2.56 % | 1.05 |
| 14 | 2,160,848 | 80,068,784 | 2.63 % | 1.06 |
| 15 | 1,766,806 | 73,468,582 | 2.35 % | 1.05 |
| 16 | 1,746,951 | 71,087,583 | 2.40 % | 1.05 |
| 17 | 1,883,533 | 68,265,948 | 2.69 % | 1.17 |
| 18 | 1,583,354 | 61,308,136 | 2.52 % | 1.05 |
| 19 | 1,568,068 | 61,903,206 | 2.47 % | 1.05 |
| 20 | 1,889,782 | 66,623,366 | 2.76 % | 1.19 |
| 21 | 1,570,588 | 61,448,017 | 2.49 % | 1.05 |
| 22 | 1,557,787 | 58,326,190 | 2.60 % | 1.06 |
| 23 | 1,296,158 | 47,362,137 | 2.66 % | 1.05 |
| 24 | 1,599,550 | 58,468,377 | 2.66 % | 1.04 |
| 25 | 1,095,105 | 41,311,112 | 2.58 % | 1.11 |
| 26 | 1,223,272 | 46,686,128 | 2.55 % | 1.04 |
| 27 | 1,331,319 | 41,931,932 | 3.08 % | 1.33 |
| 28 | 1,014,804 | 39,433,748 | 2.51 % | 1.04 |
| 29 | 1,150,897 | 43,984,994 | 2.55 % | 1.12 |
| X | 1,277,077 | 98,623,078 | 1.28 % | 1.06 |

* Depth of coverage is defined as the total sum of coverage at each covered position divided by the number of covered positions
