# Supplementary material for: A physical map of the bovine genome
Source: Genome Biol. 2007 Aug 14;8(8):R165. doi: 10.1186/gb-2007-8-8-r165 (PMC2374996; doi:10.1186/gb-2007-8-8-r165)
Supplement: Additional data file 2 — Figures showing the bovine-human comparative map defined by the BAC map and BES alignments. [file gb-2007-8-8-r165-S2.pdf]

Legend. Coverage of the bovine autosomes by fingerprint contigs and comparative mapping to the human genome (UCSC hg18 assembly). Fingerprint contigs are represented by black boxes in the middle of each chromosome panel. The first one or two digits of contig names represent the bovine chromosome number (1 to 29). The following digit indicates confidence in contig placement (0=confident; 9=uncertain). The last 2 digits indicate the relative position of the contig on the chromosome (numbered in increments of 5 to allow for future assignment of currently unanchored contigs) arranged according to BAC end sequence alignments with the UCSC hg17 assembly and the ILTX-2004 comparative map (van der Wind et al., 2004). The red bars represent the bovine composite linkage-radiation hybrid map, with marker positions indicated by red ticks. A subset of markers is displayed for reference. Grey lines represent links between BAC clones and mapped markers. Colored boxes to the left of contigs represent syntenic human genomic segments assigned to bovine chromosomes. Syntenic segments are assigned based on bovine BAC end sequence alignment against human genome sequence. Individual human chromosomes are differentially coloured. Coordinates (Mbp start and end) of each segment are indicated. White lines indicate the boundaries of syntenic segments. Boundaries are imposed where there is a change in human chromosome number, a change in orientation within the same human chromosome, or a gap greater than 10% of the last coordinate of the previous syntenic segment. This PDF document contains an oversize page with diagrams for all autosomes, and a page for each autosome.

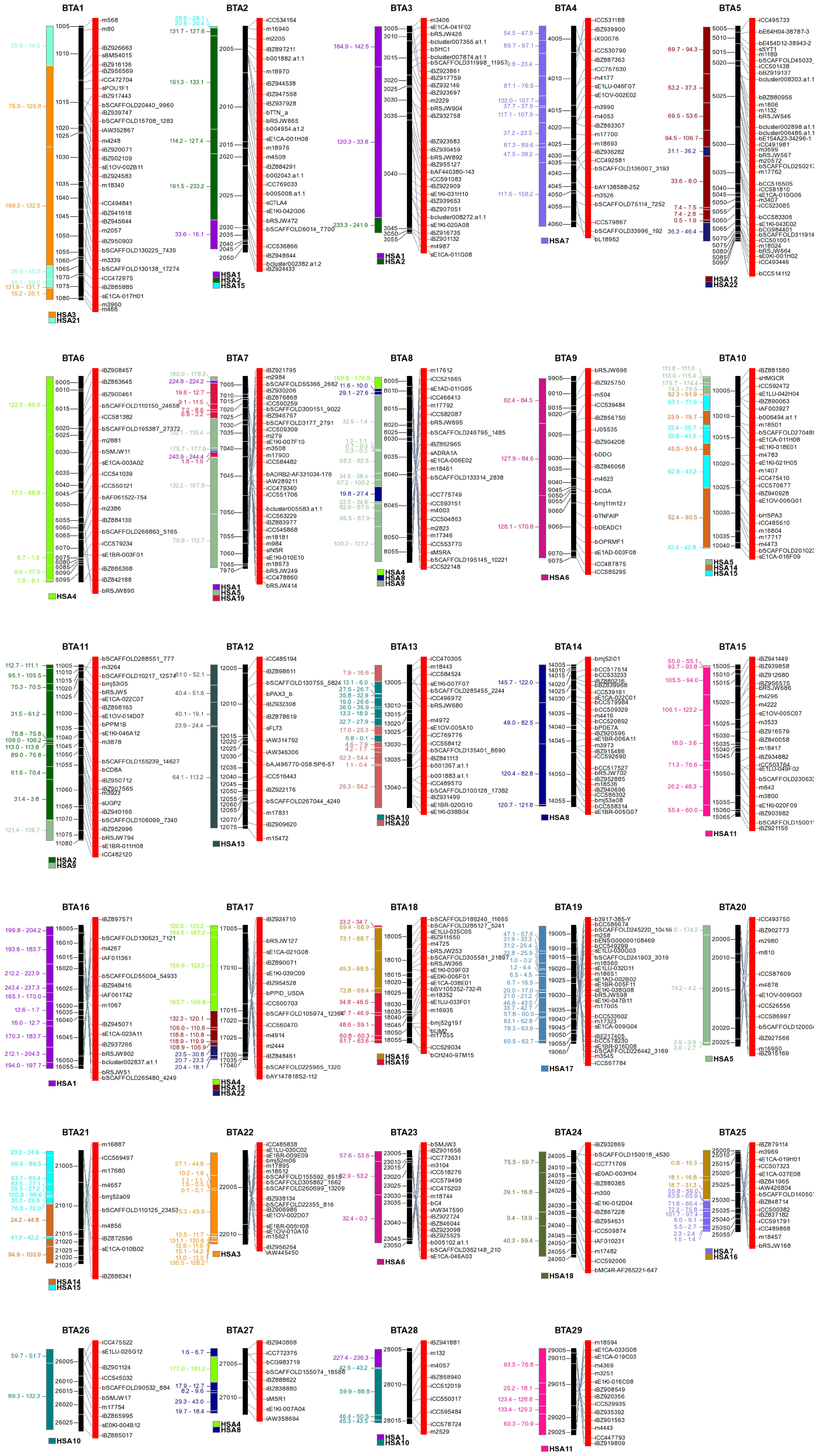

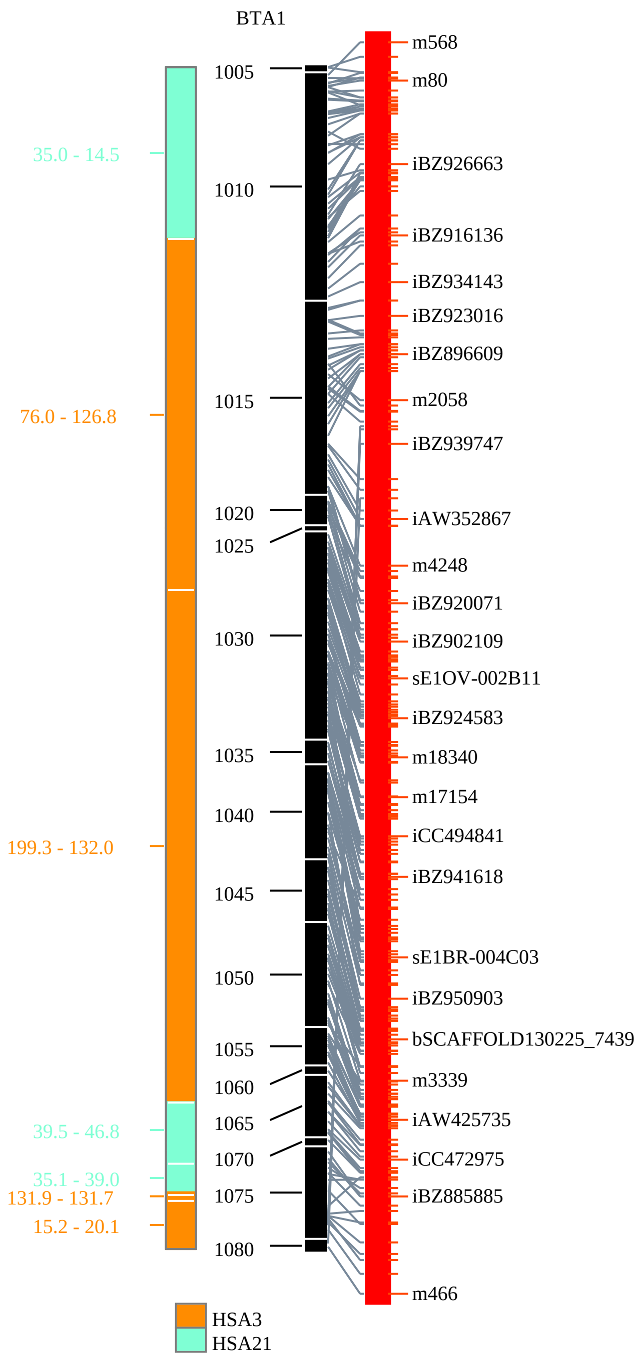
BTA1
35.0 - 14.5
76.0 - 126.8
199.3 - 132.0
39.5 - 46.8
35.1 - 39.0
131.9 - 131.7
15.2 - 20.1
1005
1010
1015
1020
1025
1030
1035
1040
1045
1050
1055
1060
1065
1070
1075
1080
m568
m80
iBZ926663
iBZ916136
iBZ934143
iBZ923016
iBZ896609
m2058
iBZ939747
iAW352867
m4248
iBZ920071
iBZ902109
sE1OV-002B11
iBZ924583
m18340
m17154
iCC494841
iBZ941618
sE1BR-004C03
iBZ950903
bSCAFFOLD130225_7439
m3339
iAW425735
iCC472975
iBZ885885
m466
HSA3
HSA21

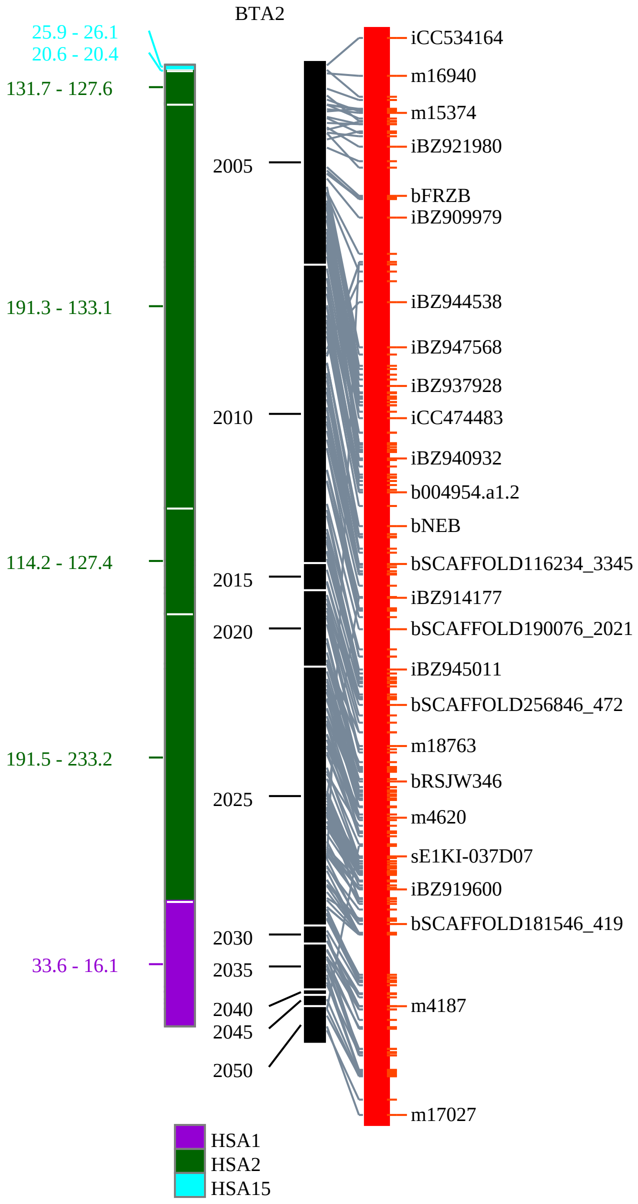

BTA2
25.9 - 26.1
20.6 - 20.4
131.7 - 127.6
191.3 - 133.1
114.2 - 127.4
191.5 - 233.2
33.6 - 16.1
2005
2010
2015
2020
2025
2030
2035
2040
2045
2050
iCC534164
m16940
m15374
iBZ921980
bFRZB
iBZ909979
iBZ944538
iBZ947568
iBZ937928
iCC474483
iBZ940932
b004954.a1.2
bNEB
bSCAFFOLD116234_3345
iBZ914177
bSCAFFOLD190076_2021
iBZ945011
bSCAFFOLD256846_472
m18763
bRSJW346
m4620
sE1KI-037D07
iBZ919600
bSCAFFOLD181546_419
m4187
m17027
HSA1
HSA2
HSA15

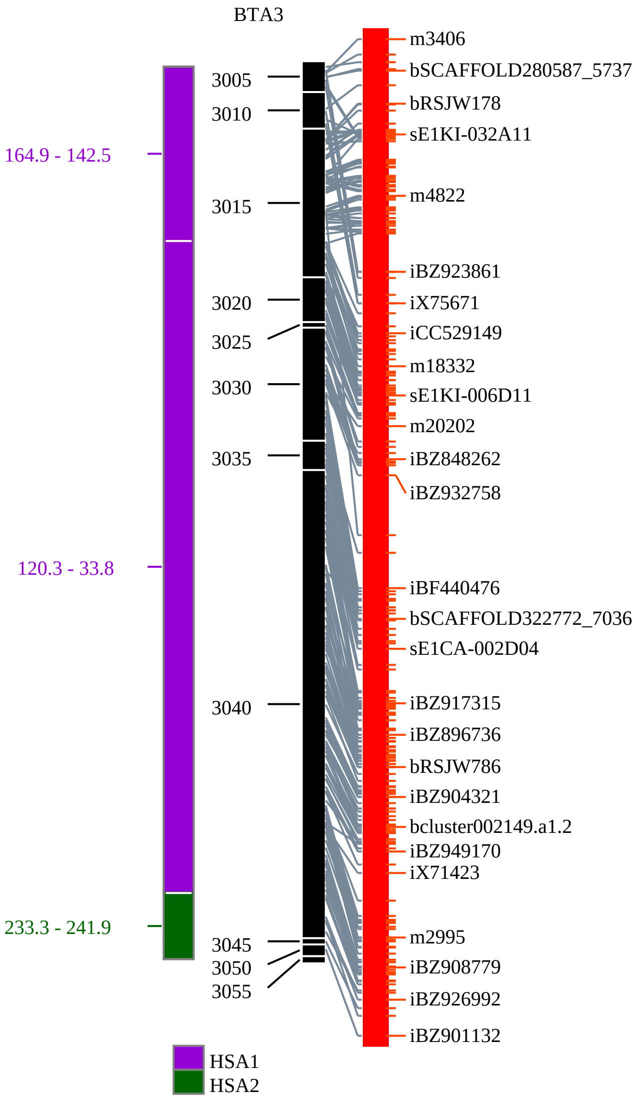
BTA3
164.9 - 142.5
120.3 - 33.8
233.3 - 241.9
3005
3010
3015
3020
3025
3030
3035
3040
3045
3050
3055
m3406
bSCAFFOLD280587_5737
bRSJW178
sE1KI-032A11
m4822
iBZ923861
iX75671
iCC529149
m18332
sE1KI-006D11
m20202
iBZ848262
iBZ932758
iBF440476
bSCAFFOLD322772_7036
sE1CA-002D04
iBZ917315
iBZ896736
bRSJW786
iBZ904321
bcluster002149.a1.2
iBZ949170
iX71423
m2995
iBZ908779
iBZ926992
iBZ901132
HSA1
HSA2

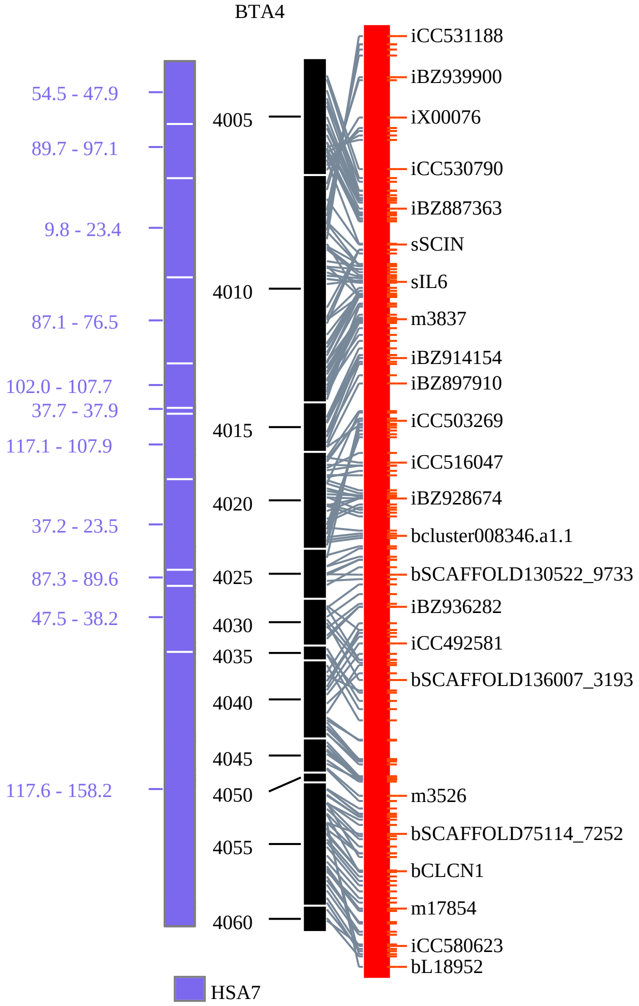

BTA4
54.5 - 47.9
89.7 - 97.1
9.8 - 23.4
87.1 - 76.5
102.0 - 107.7
37.7 - 37.9
117.1 - 107.9
37.2 - 23.5
87.3 - 89.6
47.5 - 38.2
117.6 - 158.2
4005
4010
4015
4020
4025
4030
4035
4040
4045
4050
4055
4060
iCC531188
iBZ939900
iX00076
iCC530790
iBZ887363
sSCIN
sIL6
m3837
iBZ914154
iBZ897910
iCC503269
iCC516047
iBZ928674
bcluster008346.a1.1
bSCAFFOLD130522_9733
iBZ936282
iCC492581
bSCAFFOLD136007_3193
m3526
bSCAFFOLD75114_7252
bCLCN1
m17854
iCC580623
bL18952
HSA7

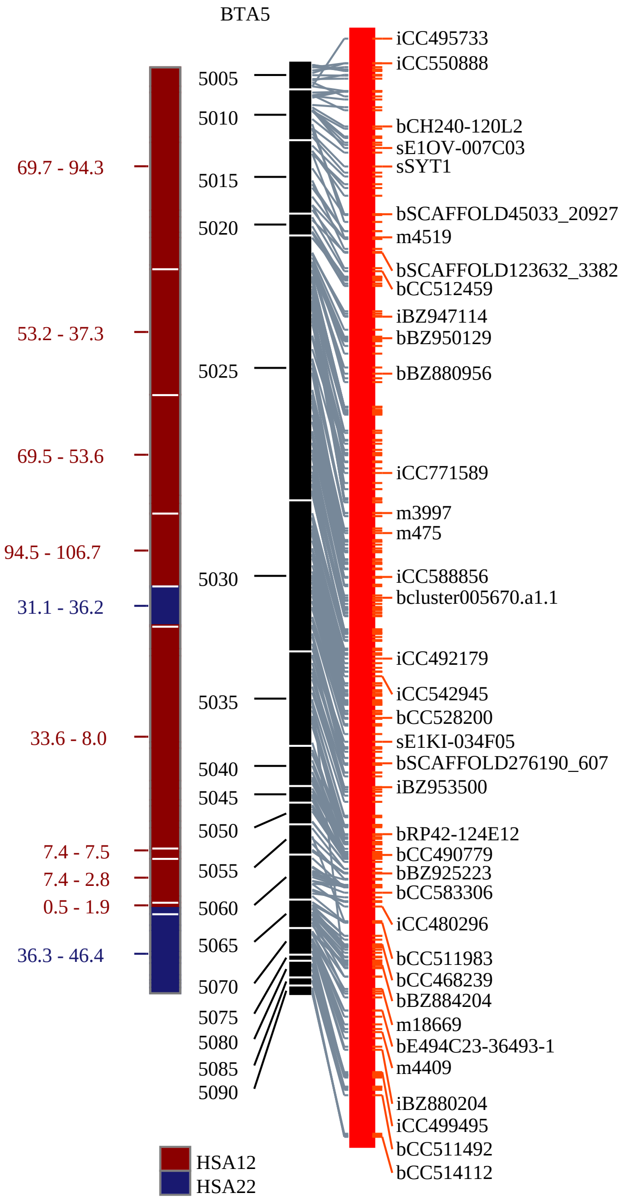
BTA5
69.7 - 94.3
53.2 - 37.3
69.5 - 53.6
94.5 - 106.7
31.1 - 36.2
33.6 - 8.0
7.4 - 7.5
7.4 - 2.8
0.5 - 1.9
36.3 - 46.4
5005
5010
5015
5020
5025
5030
5035
5040
5045
5050
5055
5060
5065
5070
5075
5080
5085
5090
iCC495733
iCC550888
bCH240-120L2
sE1OV-007C03
sSYT1
bSCAFFOLD45033_20927
m4519
bSCAFFOLD123632_3382
bCC512459
iBZ947114
bBZ950129
bBZ880956
iCC771589
m3997
m475
iCC588856
bcluster005670.a1.1
iCC492179
iCC542945
bCC528200
sE1KI-034F05
bSCAFFOLD276190_607
iBZ953500
bRP42-124E12
bCC490779
bBZ925223
bCC583306
iCC480296
bCC511983
bCC468239
bBZ884204
m18669
bE494C23-36493-1
m4409
iBZ880204
iCC499495
bCC511492
bCC514112
HSA12
HSA22

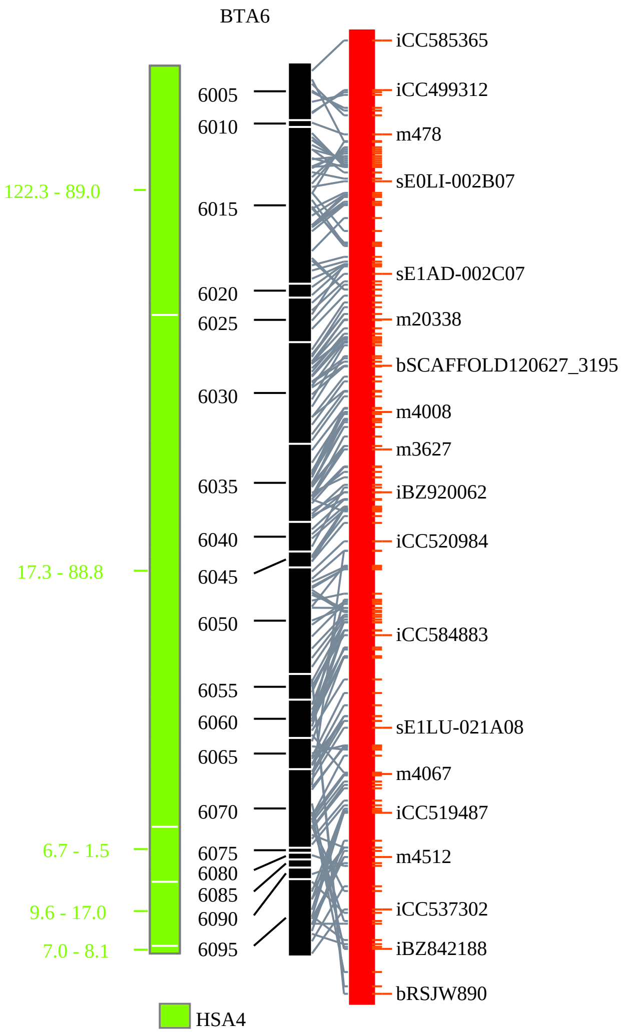

BTA6
122.3 - 89.0
17.3 - 88.8
6.7 - 1.5
9.6 - 17.0
7.0 - 8.1
6005
6010
6015
6020
6025
6030
6035
6040
6045
6050
6055
6060
6065
6070
6075
6080
6085
6090
6095
iCC585365
iCC499312
m478
sE0LI-002B07
sE1AD-002C07
m20338
bSCAFFOLD120627_3195
m4008
m3627
iBZ920062
iCC520984
iCC584883
sE1LU-021A08
m4067
iCC519487
m4512
iCC537302
iBZ842188
bRSJW890
HSA4

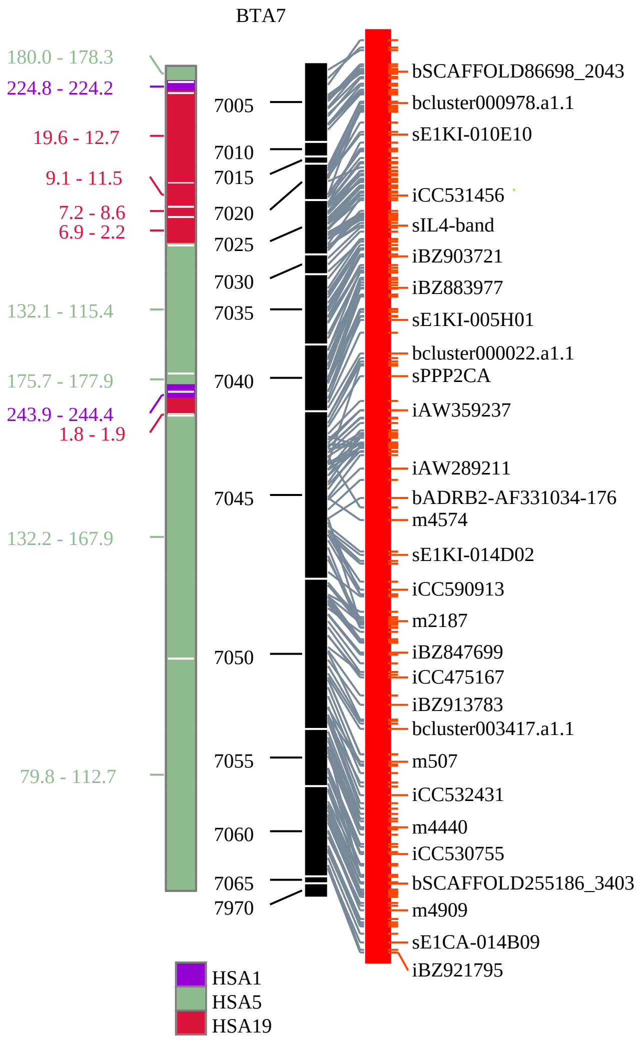
BTA7
180.0 - 178.3
224.8 - 224.2
19.6 - 12.7
9.1 - 11.5
7.2 - 8.6
6.9 - 2.2
132.1 - 115.4
175.7 - 177.9
243.9 - 244.4
1.8 - 1.9
132.2 - 167.9
79.8 - 112.7
7005
7010
7015
7020
7025
7030
7035
7040
7045
7050
7055
7060
7065
7970
bSCAFFOLD86698_2043
bcluster000978.a1.1
sE1KI-010E10
iCC531456
sIL4-band
iBZ903721
iBZ883977
sE1KI-005H01
bcluster000022.a1.1
sPPP2CA
iAW359237
iAW289211
bADRB2-AF331034-176
m4574
sE1KI-014D02
iCC590913
m2187
iBZ847699
iCC475167
iBZ913783
bcluster003417.a1.1
m507
iCC532431
m4440
iCC530755
bSCAFFOLD255186_3403
m4909
sE1CA-014B09
iBZ921795
HSA1
HSA5
HSA19

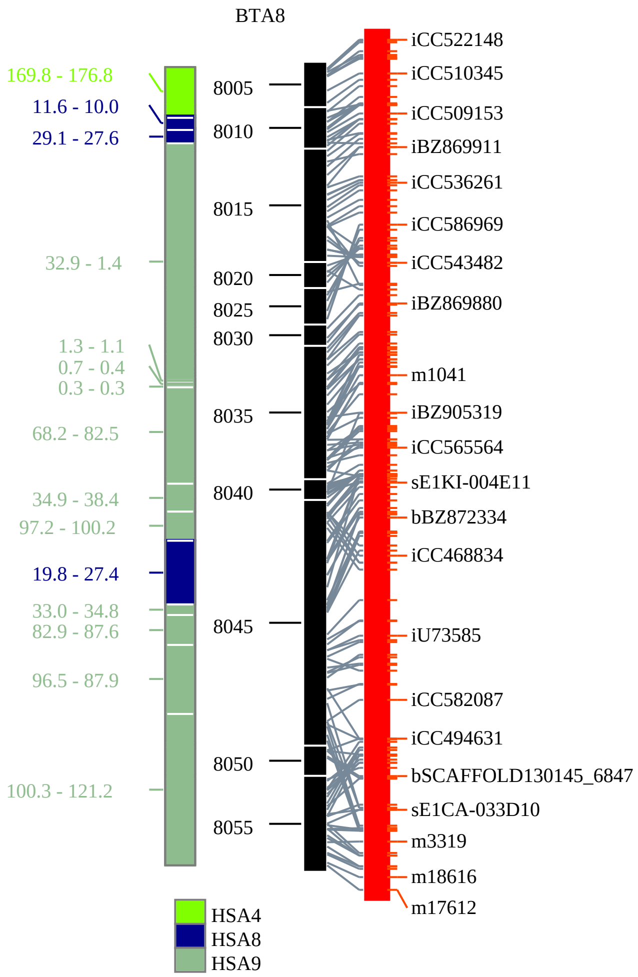

BTA8
169.8 - 176.8
11.6 - 10.0
29.1 - 27.6
32.9 - 1.4
1.3 - 1.1
0.7 - 0.4
0.3 - 0.3
68.2 - 82.5
34.9 - 38.4
97.2 - 100.2
19.8 - 27.4
33.0 - 34.8
82.9 - 87.6
96.5 - 87.9
100.3 - 121.2
8005
8010
8015
8020
8025
8030
8035
8040
8045
8050
8055
iCC522148
iCC510345
iCC509153
iBZ869911
iCC536261
iCC586969
iCC543482
iBZ869880
m1041
iBZ905319
iCC565564
sE1KI-004E11
bBZ872334
iCC468834
iU73585
iCC582087
iCC494631
bSCAFFOLD130145_6847
sE1CA-033D10
m3319
m18616
m17612
HSA4
HSA8
HSA9

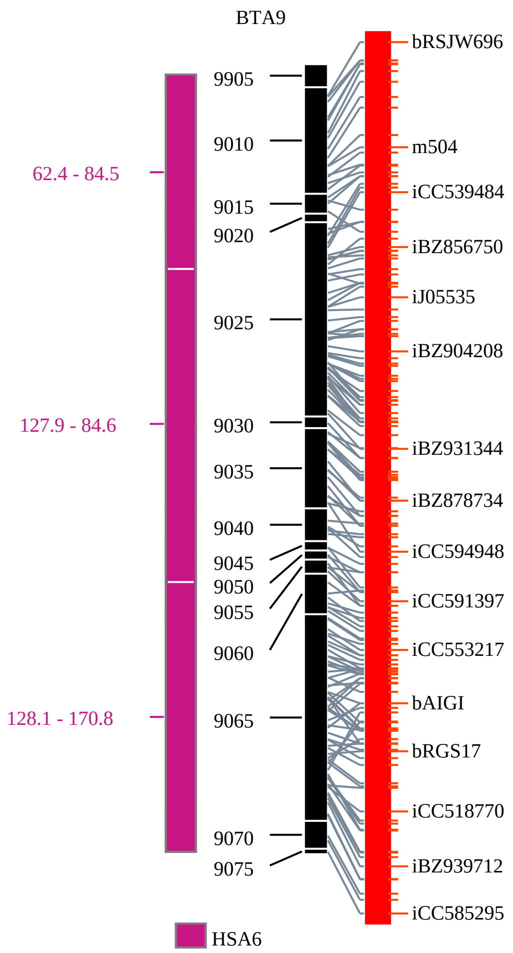

BTA9
62.4 - 84.5
127.9 - 84.6
128.1 - 170.8
9905
9010
9015
9020
9025
9030
9035
9040
9045
9050
9055
9060
9065
9070
9075
bRSJW696
m504
iCC539484
iBZ856750
iJ05535
iBZ904208
iBZ931344
iBZ878734
iCC594948
iCC591397
iCC553217
bAIGI
bRGS17
iCC518770
iBZ939712
iCC585295
HSA6

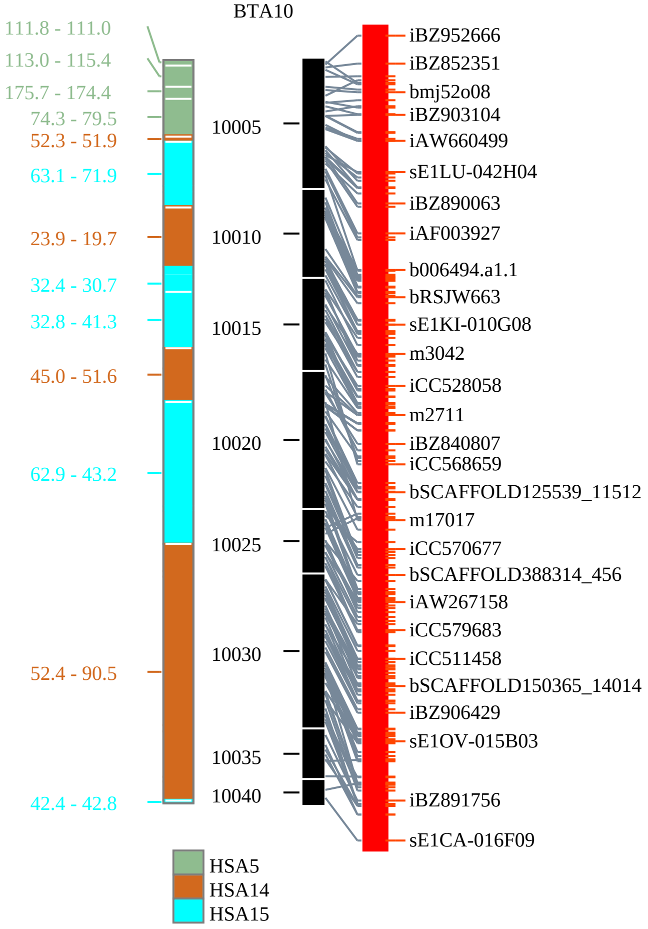

BTA10
111.8 - 111.0
113.0 - 115.4
175.7 - 174.4
74.3 - 79.5
52.3 - 51.9
63.1 - 71.9
23.9 - 19.7
32.4 - 30.7
32.8 - 41.3
45.0 - 51.6
62.9 - 43.2
52.4 - 90.5
42.4 - 42.8
10005
10010
10015
10020
10025
10030
10035
10040
iBZ952666
iBZ852351
bmj52o08
iBZ903104
iAW660499
sE1LU-042H04
iBZ890063
iAF003927
b006494.a1.1
bRSJW663
sE1KI-010G08
m3042
iCC528058
m2711
iBZ840807
iCC568659
bSCAFFOLD125539_11512
m17017
iCC570677
bSCAFFOLD388314_456
iAW267158
iCC579683
iCC511458
bSCAFFOLD150365_14014
iBZ906429
sE1OV-015B03
iBZ891756
sE1CA-016F09
HSA5
HSA14
HSA15

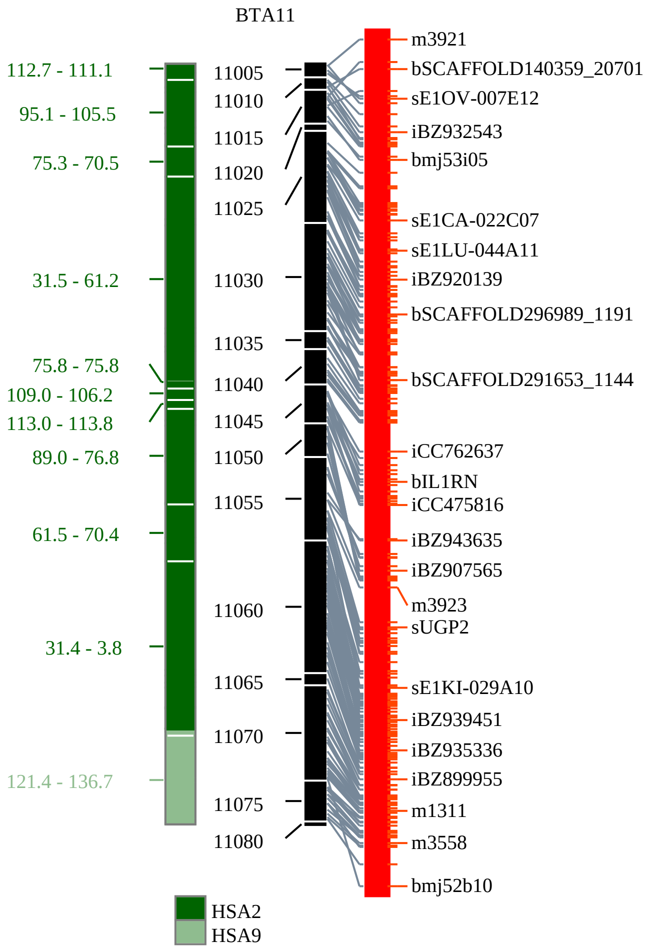
BTA11
112.7 - 111.1
95.1 - 105.5
75.3 - 70.5
31.5 - 61.2
75.8 - 75.8
109.0 - 106.2
113.0 - 113.8
89.0 - 76.8
61.5 - 70.4
31.4 - 3.8
121.4 - 136.7
11005
11010
11015
11020
11025
11030
11035
11040
11045
11050
11055
11060
11065
11070
11075
11080
m3921
bSCAFFOLD140359_20701
sE1OV-007E12
iBZ932543
bmj53i05
sE1CA-022C07
sE1LU-044A11
iBZ920139
bSCAFFOLD296989_1191
bSCAFFOLD291653_1144
iCC762637
bIL1RN
iCC475816
iBZ943635
iBZ907565
m3923
sUGP2
sE1KI-029A10
iBZ939451
iBZ935336
iBZ899955
m1311
m3558
bmj52b10
HSA2
HSA9

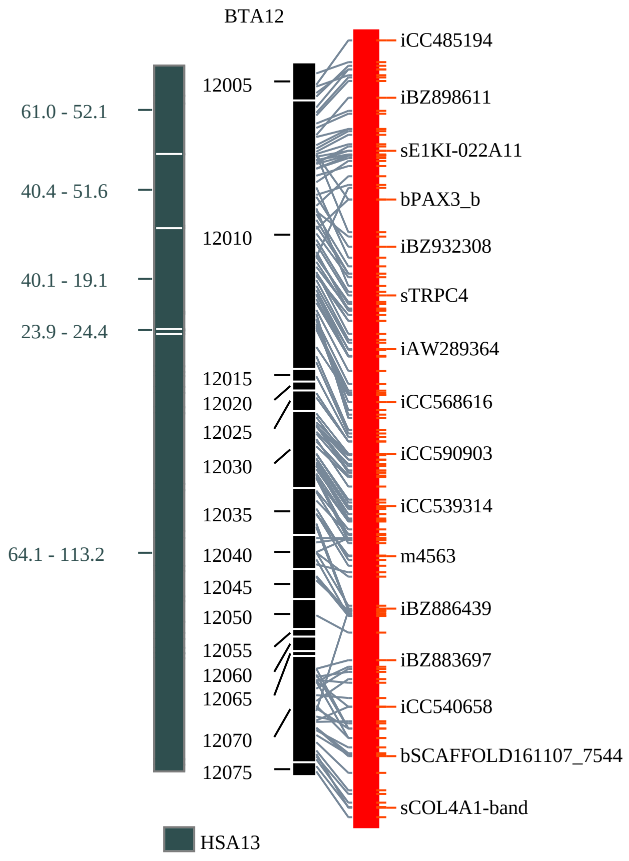
BTA12
61.0 - 52.1
40.4 - 51.6
40.1 - 19.1
23.9 - 24.4
64.1 - 113.2
12005
12010
12015
12020
12025
12030
12035
12040
12045
12050
12055
12060
12065
12070
12075
iCC485194
iBZ898611
sE1KI-022A11
bPAX3_b
iBZ932308
sTRPC4
iAW289364
iCC568616
iCC590903
iCC539314
m4563
iBZ886439
iBZ883697
iCC540658
bSCAFFOLD161107_7544
sCOL4A1-band
HSA13

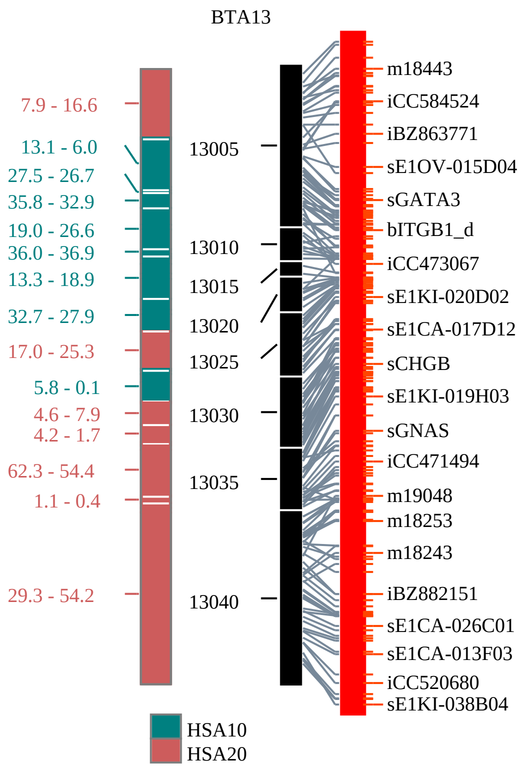

BTA13
7.9 - 16.6
13.1 - 6.0
27.5 - 26.7
35.8 - 32.9
19.0 - 26.6
36.0 - 36.9
13.3 - 18.9
32.7 - 27.9
17.0 - 25.3
5.8 - 0.1
4.6 - 7.9
4.2 - 1.7
62.3 - 54.4
1.1 - 0.4
29.3 - 54.2
13005
13010
13015
13020
13025
13030
13035
13040
m18443
iCC584524
iBZ863771
sE1OV-015D04
sGATA3
bITGB1_d
iCC473067
sE1KI-020D02
sE1CA-017D12
sCHGB
sE1KI-019H03
sGNAS
iCC471494
m19048
m18253
m18243
iBZ882151
sE1CA-026C01
sE1CA-013F03
iCC520680
sE1KI-038B04
HSA10
HSA20

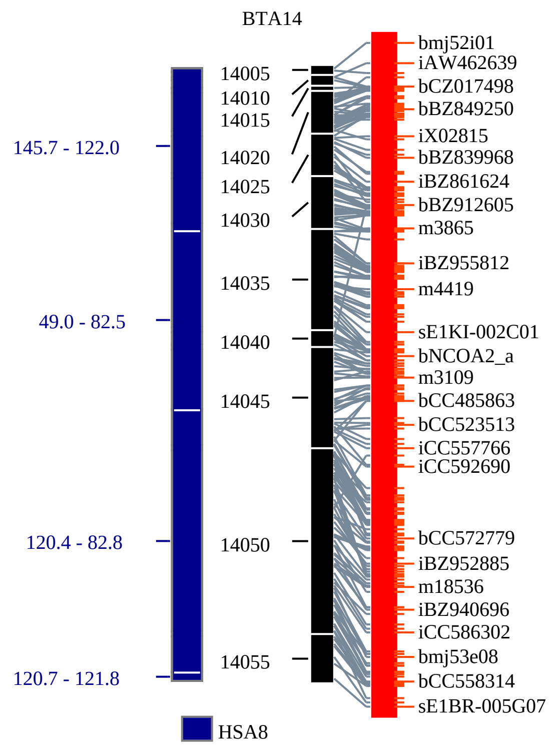
BTA14
145.7 - 122.0
49.0 - 82.5
120.4 - 82.8
120.7 - 121.8
HSA8
14005
14010
14015
14020
14025
14030
14035
14040
14045
14050
14055
bmj52i01
iAW462639
bCZ017498
bBZ849250
iX02815
bBZ839968
iBZ861624
bBZ912605
m3865
iBZ955812
m4419
sE1KI-002C01
bNCOA2_a
m3109
bCC485863
bCC523513
iCC557766
iCC592690
bCC572779
iBZ952885
m18536
iBZ940696
iCC586302
bmj53e08
bCC558314
sE1BR-005G07

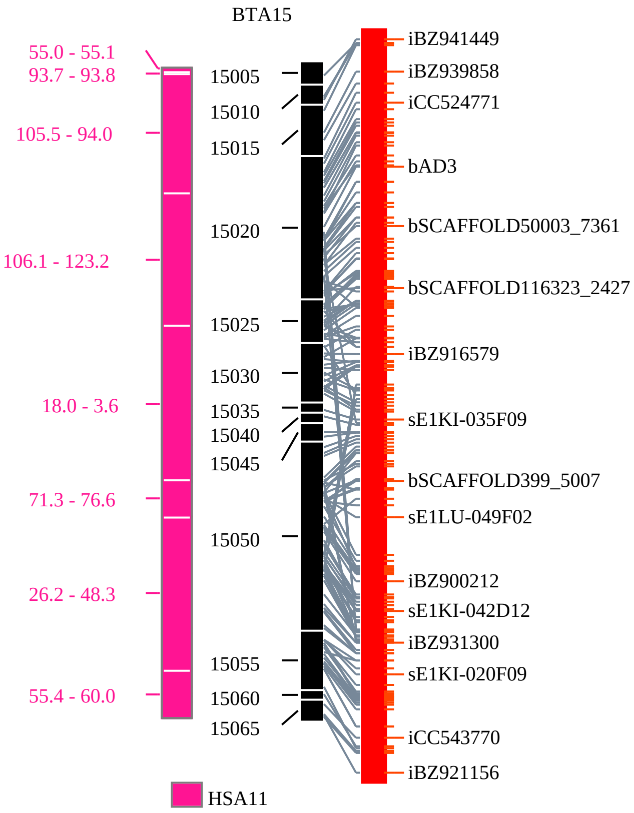

BTA15
55.0 - 55.1
93.7 - 93.8
105.5 - 94.0
106.1 - 123.2
18.0 - 3.6
71.3 - 76.6
26.2 - 48.3
55.4 - 60.0
15005
15010
15015
15020
15025
15030
15035
15040
15045
15050
15055
15060
15065
iBZ941449
iBZ939858
iCC524771
bAD3
bSCAFFOLD50003_7361
bSCAFFOLD116323_2427
iBZ916579
sE1KI-035F09
bSCAFFOLD399_5007
sE1LU-049F02
iBZ900212
sE1KI-042D12
iBZ931300
sE1KI-020F09
iCC543770
iBZ921156
HSA11

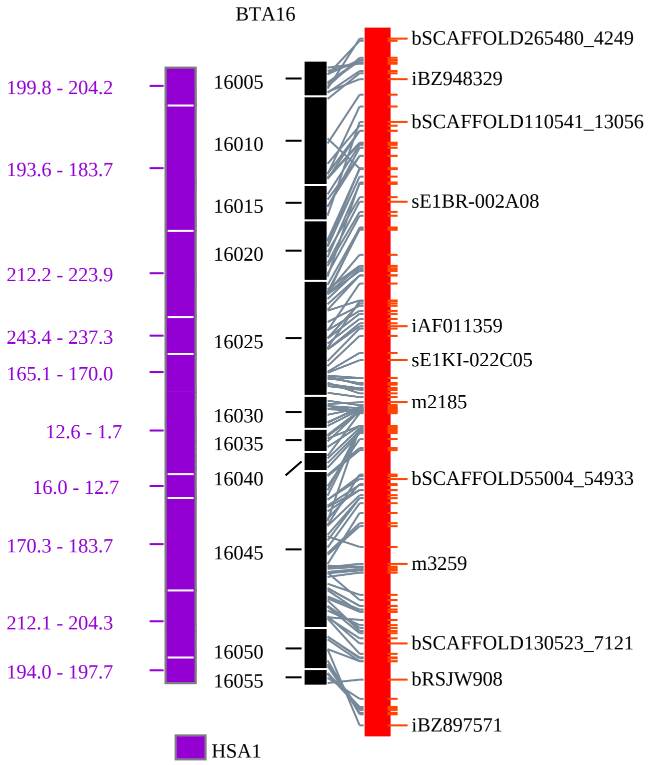

BTA16
199.8 - 204.2
193.6 - 183.7
212.2 - 223.9
243.4 - 237.3
165.1 - 170.0
12.6 - 1.7
16.0 - 12.7
170.3 - 183.7
212.1 - 204.3
194.0 - 197.7
16005
16010
16015
16020
16025
16030
16035
16040
16045
16050
16055
bSCAFFOLD265480_4249
iBZ948329
bSCAFFOLD110541_13056
sE1BR-002A08
iAF011359
sE1KI-022C05
m2185
bSCAFFOLD55004_54933
m3259
bSCAFFOLD130523_7121
bRSJW908
iBZ897571
HSA1

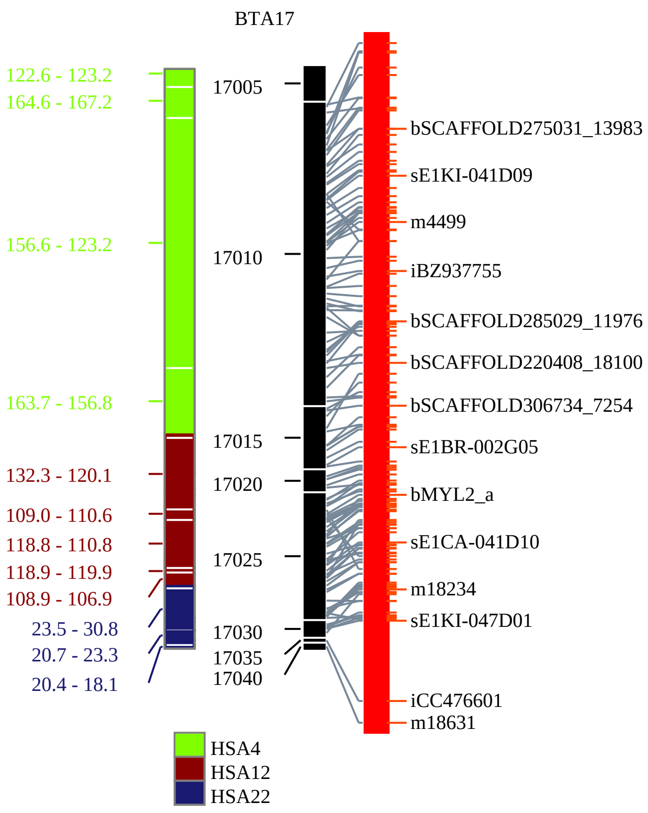
BTA17
122.6 - 123.2
164.6 - 167.2
156.6 - 123.2
163.7 - 156.8
132.3 - 120.1
109.0 - 110.6
118.8 - 110.8
118.9 - 119.9
108.9 - 106.9
23.5 - 30.8
20.7 - 23.3
20.4 - 18.1
17005
17010
17015
17020
17025
17030
17035
17040
bSCAFFOLD275031_13983
sE1KI-041D09
m4499
iBZ937755
bSCAFFOLD285029_11976
bSCAFFOLD220408_18100
bSCAFFOLD306734_7254
sE1BR-002G05
bMYL2_a
sE1CA-041D10
m18234
sE1KI-047D01
iCC476601
m18631
HSA4
HSA12
HSA22

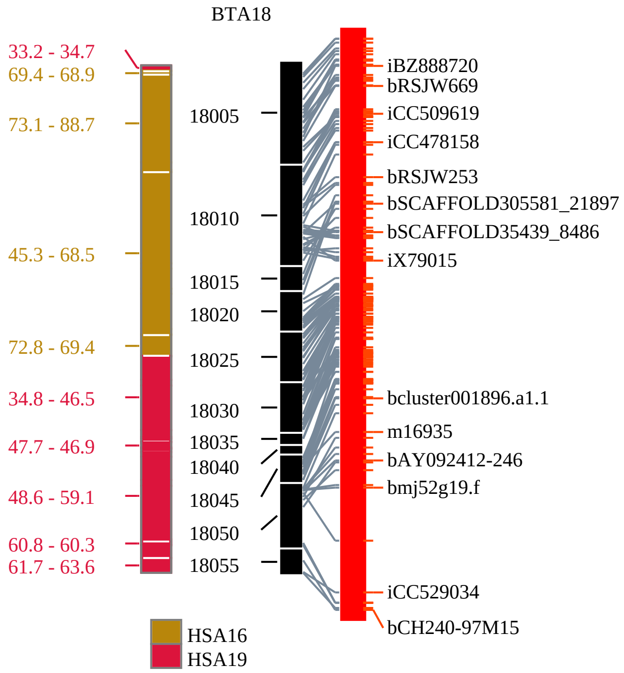

BTA18
33.2 - 34.7
69.4 - 68.9
73.1 - 88.7
45.3 - 68.5
72.8 - 69.4
34.8 - 46.5
47.7 - 46.9
48.6 - 59.1
60.8 - 60.3
61.7 - 63.6
18005
18010
18015
18020
18025
18030
18035
18040
18045
18050
18055
iBZ888720
bRSJW669
iCC509619
iCC478158
bRSJW253
bSCAFFOLD305581_21897
bSCAFFOLD35439_8486
iX79015
bcluster001896.a1.1
m16935
bAY092412-246
bmj52g19.f
iCC529034
bCH240-97M15
HSA16
HSA19

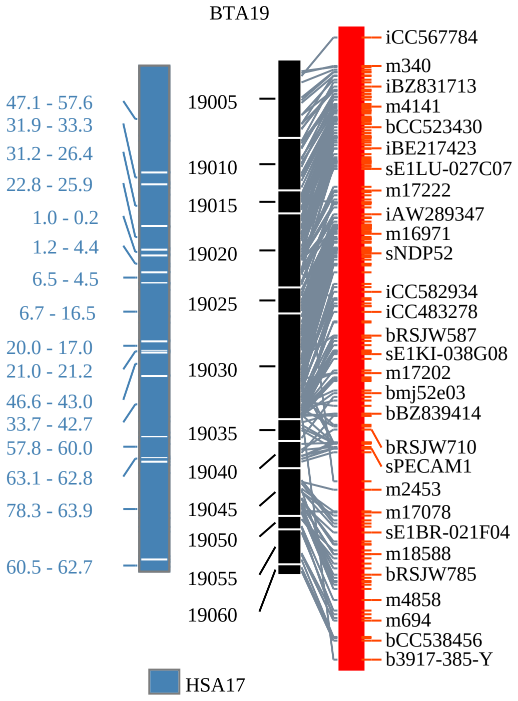

BTA19
47.1 - 57.6
31.9 - 33.3
31.2 - 26.4
22.8 - 25.9
1.0 - 0.2
1.2 - 4.4
6.5 - 4.5
6.7 - 16.5
20.0 - 17.0
21.0 - 21.2
46.6 - 43.0
33.7 - 42.7
57.8 - 60.0
63.1 - 62.8
78.3 - 63.9
60.5 - 62.7
19005
19010
19015
19020
19025
19030
19035
19040
19045
19050
19055
19060
iCC567784
m340
iBZ831713
m4141
bCC523430
iBE217423
sE1LU-027C07
m17222
iAW289347
m16971
sNDP52
iCC582934
iCC483278
bRSJW587
sE1KI-038G08
m17202
bmj52e03
bBZ839414
bRSJW710
sPECAM1
m2453
m17078
sE1BR-021F04
m18588
bRSJW785
m4858
m694
bCC538456
b3917-385-Y
HSA17

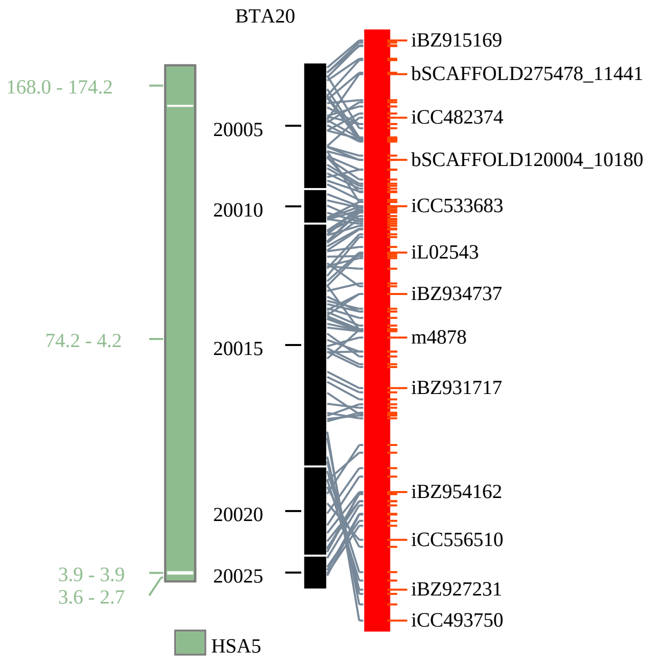
BTA20
168.0 - 174.2
74.2 - 4.2
3.9 - 3.9
3.6 - 2.7
20005
20010
20015
20020
20025
iBZ915169
bSCAFFOLD275478_11441
iCC482374
bSCAFFOLD120004_10180
iCC533683
iL02543
iBZ934737
m4878
iBZ931717
iBZ954162
iCC556510
iBZ927231
iCC493750
HSA5

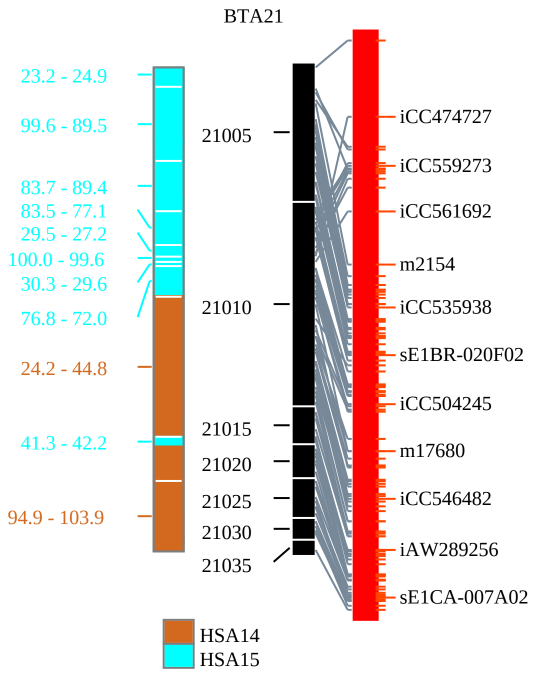
BTA21
23.2 - 24.9
99.6 - 89.5
83.7 - 89.4
83.5 - 77.1
29.5 - 27.2
100.0 - 99.6
30.3 - 29.6
76.8 - 72.0
24.2 - 44.8
41.3 - 42.2
94.9 - 103.9
21005
21010
21015
21020
21025
21030
21035
iCC474727
iCC559273
iCC561692
m2154
iCC535938
sE1BR-020F02
iCC504245
m17680
iCC546482
iAW289256
sE1CA-007A02
HSA14
HSA15

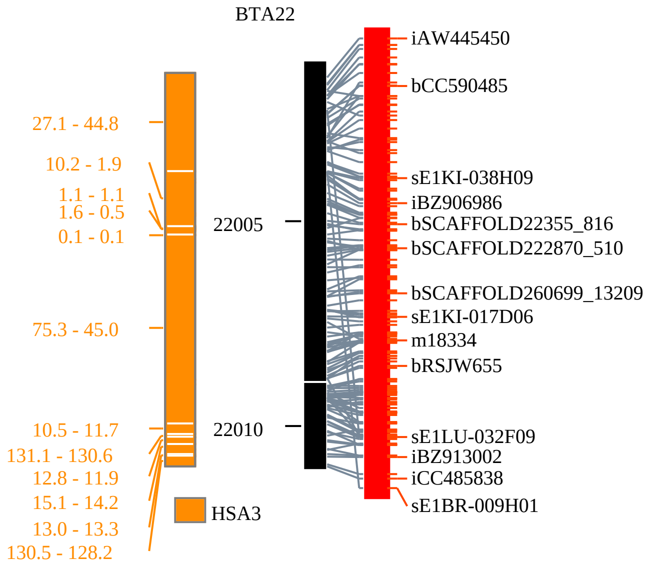

BTA22
iAW445450
bCC590485
sE1KI-038H09
iBZ906986
bSCAFFOLD22355_816
bSCAFFOLD222870_510
bSCAFFOLD260699_13209
sE1KI-017D06
m18334
bRSJW655
sE1LU-032F09
iBZ913002
iCC485838
sE1BR-009H01
22005
22010
27.1 - 44.8
10.2 - 1.9
1.1 - 1.1
1.6 - 0.5
0.1 - 0.1
75.3 - 45.0
10.5 - 11.7
131.1 - 130.6
12.8 - 11.9
15.1 - 14.2
13.0 - 13.3
130.5 - 128.2
HSA3

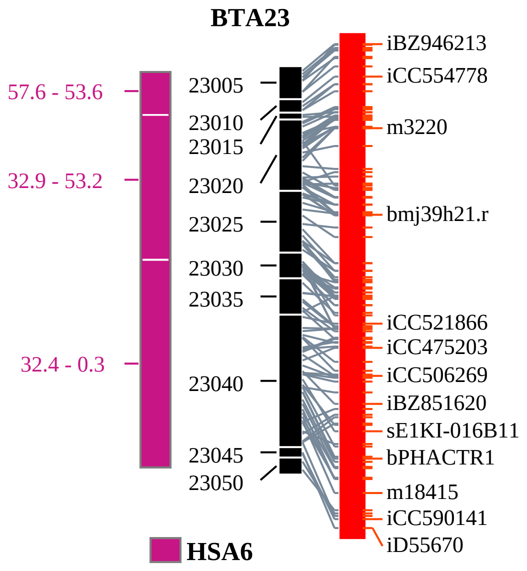
BTA23
57.6 - 53.6
32.9 - 53.2
32.4 - 0.3
23005
23010
23015
23020
23025
23030
23035
23040
23045
23050
iBZ946213
iCC554778
m3220
bmj39h21.r
iCC521866
iCC475203
iCC506269
iBZ851620
sE1KI-016B11
bPHACTR1
m18415
iCC590141
iD55670
HSA6

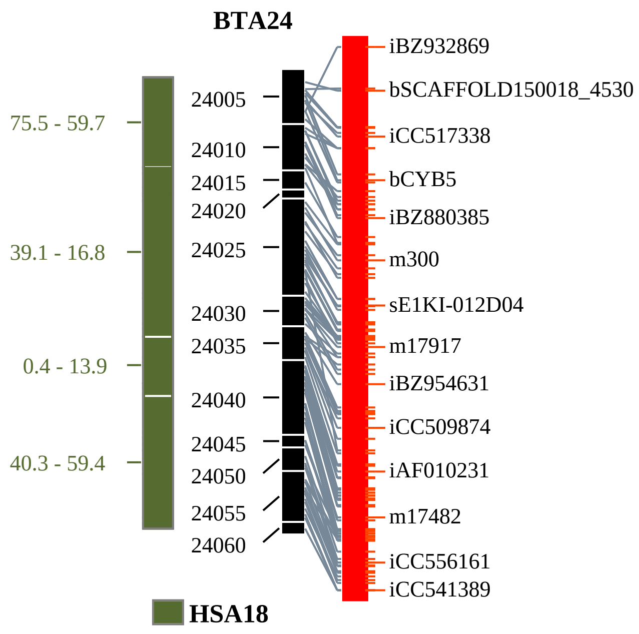

BTA24
75.5 - 59.7
39.1 - 16.8
0.4 - 13.9
40.3 - 59.4
24005
24010
24015
24020
24025
24030
24035
24040
24045
24050
24055
24060
iBZ932869
bSCAFFOLD150018_4530
iCC517338
bCYB5
iBZ880385
m300
sE1KI-012D04
m17917
iBZ954631
iCC509874
iAF010231
m17482
iCC556161
iCC541389
HSA18

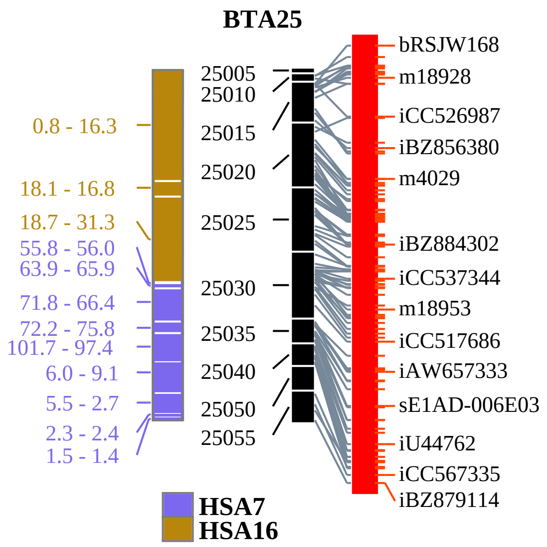
BTA25
0.8 - 16.3
18.1 - 16.8
18.7 - 31.3
55.8 - 56.0
63.9 - 65.9
71.8 - 66.4
72.2 - 75.8
101.7 - 97.4
6.0 - 9.1
5.5 - 2.7
2.3 - 2.4
1.5 - 1.4
25005
25010
25015
25020
25025
25030
25035
25040
25050
25055
bRSJW168
m18928
iCC526987
iBZ856380
m4029
iBZ884302
iCC537344
m18953
iCC517686
iAW657333
sE1AD-006E03
iU44762
iCC567335
iBZ879114
HSA7
HSA16

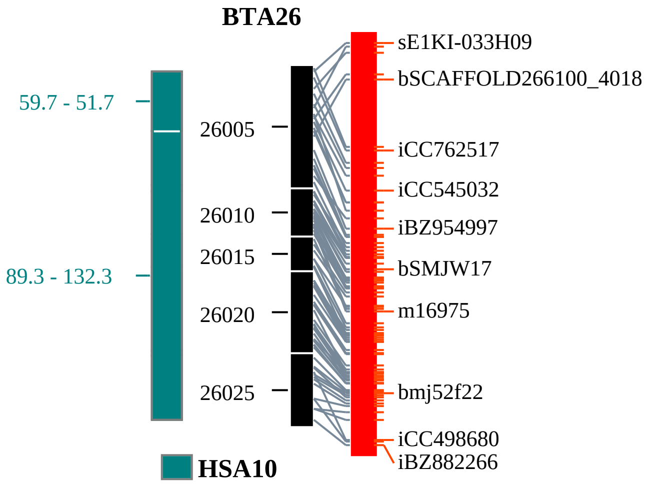
BTA26
59.7 - 51.7
89.3 - 132.3
26005
26010
26015
26020
26025
sE1KI-033H09
bSCAFFOLD266100_4018
iCC762517
iCC545032
iBZ954997
bSMJW17
m16975
bmj52f22
iCC498680
iBZ882266
HSA10

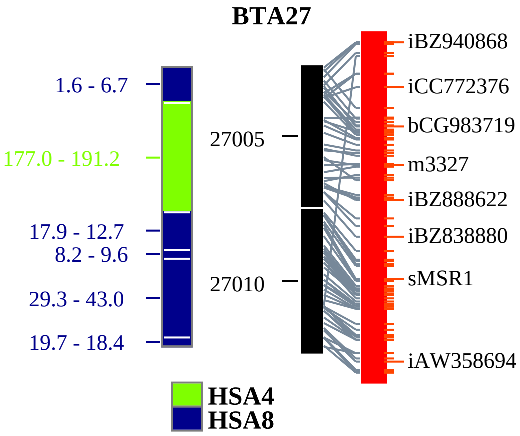
BTA27
1.6 - 6.7
177.0 - 191.2
17.9 - 12.7
8.2 - 9.6
29.3 - 43.0
19.7 - 18.4
HSA4
HSA8
27005
27010
iBZ940868
iCC772376
bCG983719
m3327
iBZ888622
iBZ838880
sMSR1
iAW358694

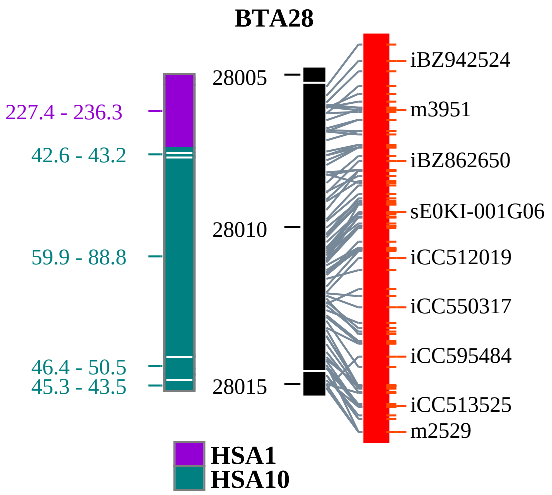
BTA28
227.4 - 236.3
42.6 - 43.2
59.9 - 88.8
46.4 - 50.5
45.3 - 43.5
28005
28010
28015
iBZ942524
m3951
iBZ862650
sE0KI-001G06
iCC512019
iCC550317
iCC595484
iCC513525
m2529
HSA1
HSA10

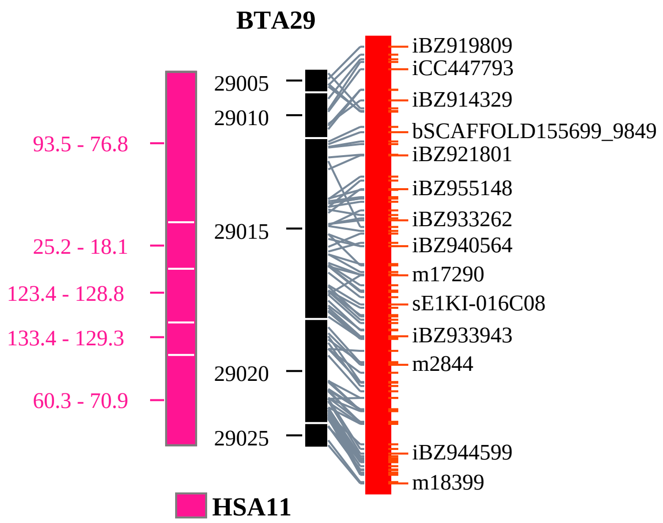

BTA29
93.5 - 76.8
25.2 - 18.1
123.4 - 128.8
133.4 - 129.3
60.3 - 70.9
29005
29010
29015
29020
29025
iBZ919809
iCC447793
iBZ914329
bSCAFFOLD155699_9849
iBZ921801
iBZ955148
iBZ933262
iBZ940564
m17290
sE1KI-016C08
iBZ933943
m2844
iBZ944599
m18399
HSA11
